# PSTVd infection in *Nicotiana benthamiana* plants has a minor yet detectable effect on CG methylation

**DOI:** 10.3389/fpls.2023.1258023

**Published:** 2023-10-31

**Authors:** Martha Tselika, Nektarios Belmezos, Paraskevi Kallemi, Christos Andronis, Michela Chiumenti, Beatriz Navarro, Matthieu Lavigne, Francesco Di Serio, Kriton Kalantidis, Konstantina Katsarou

**Affiliations:** ^1^ Department of Biology, University of Crete, Heraklion, Crete, Greece; ^2^ Institute of Molecular Biology and Biotechnology, Foundation for Research and Technology-Hellas, Heraklion, Crete, Greece; ^3^ Istituto per la Protezione Sostenibile delle Piante, Consiglio Nazionale delle Ricerche, Bari, Italy

**Keywords:** chop PCR, MRSE, MSAP-Seq, bisulfite sequencing, potato spindle tuber viroid

## Abstract

Viroids are small circular RNAs infecting a wide range of plants. They do not code for any protein or peptide and therefore rely on their structure for their biological cycle. Observed phenotypes of viroid infected plants are thought to occur through changes at the transcriptional/translational level of the host. A mechanism involved in such changes is RNA-directed DNA methylation (RdDM). Till today, there are contradictory works about viroids interference of RdDM. In this study, we investigated the epigenetic effect of viroid infection in *Nicotiana benthamiana* plants. Using potato spindle tuber viroid (PSTVd) as the triggering pathogen and via bioinformatic analyses, we identified endogenous gene promoters and transposable elements targeted by 24 nt host siRNAs that differentially accumulated in PSTVd-infected and healthy plants. The methylation status of these targets was evaluated following digestion with methylation-sensitive restriction enzymes coupled with PCR amplification, and bisulfite sequencing. In addition, we used Methylation Sensitive Amplification Polymorphism (MSAP) followed by sequencing (MSAP-seq) to study genomic DNA methylation of 5-methylcytosine (^5m^C) in CG sites upon viroid infection. In this study we identified a limited number of target loci differentially methylated upon PSTVd infection. These results enhance our understanding of the epigenetic host changes as a result of pospiviroid infection.

## 1 Introduction

DNA methylation is an epigenetic mechanism that regulates gene expression, imprinting and genome stability. In plants DNA methylation occurs at position 5 (^5m^C) of CG, CHG or CHH (H represents A, T or C) context (Erdmann and Picard, 2020; Gallego-Bartolome, 2020; Kryovrysanaki et al., 2022). CG is the predominant site for methylation, followed by CHG and CHH. *Arabidopsis thaliana* (*A. thaliana*) in canonical growth conditions displays methylation levels of approximately 24% for CG, 6.7% for CHC and 1.7% for CHH (Cokus et al., 2008), whereas in *Oryza sativa* a three times larger genome, 59.4% of CG were found methylated, followed by 20.7% of CHG and 2.18% of CHH (Feng et al., 2010). In *Zea may*s genome, 86% CG, 74% CHG, and 5.4% CHH were found methylated (Gent et al., 2013). The difference in percentages at each sequence between these three plants suggests that methylation depends on species.

RNA-DIRECTED DNA METHYLATION (RdDM) is known to regulate DNA methylation via at least two separate pathways. The canonical pathway involves specific proteins, called “histone readers”, which recruit RNA POLYMERASE IV (Pol IV) to the chromatin. Pol IV synthesizes single-stranded RNAs (ssRNAs) that are taken over by RNA-DEPENDENT RNA POLYMERASE 2 (RdR2), and double-stranded RNAs (dsRNAs) are generated and diced by DICER-LIKE PROTEIN 3 (DCL3) producing 24 nt small interfering RNAs (siRNAs). To avoid degradation, the 24 nt siRNAs are 3’ methylated by HUA ENHANCER 1 (HEN1) before being incorporated into a complex consisting (in *A. thaliana*) of ARGONAUTE 4 (AGO4) and ARGONAUTE 6 (AGO6) proteins. These siRNAs bind to non-coding RNAs (ncRNAs) produced by RNA POLYMERASE V (Pol V) by complementarity. Once the complex AGO4/6-siRNA-ncRNA-PolV is formed, the DNA methyltransferase DOMAINS REARRANGED METHYLTRANSFERASE 2 (DRM2) catalyzes *de novo* DNA methylation (Zhang et al., 2018; Erdmann and Picard, 2020; Gallego-Bartolome, 2020; Kryovrysanaki et al., 2022). The second pathway is the non-canonical and is triggered by siRNAs produced by dsRNAs of different origins (e.g. viruses, Pol II transcripts) and sizes (21-24 nt formed by distinct DCL proteins). These siRNAs are loaded with AGO4 and/or AGO6 and trigger the DRM2-dependent methylation (Cuerda-Gil and Slotkin, 2016).

Following establishment, methylation can be maintained through specific protein pathways. CG methylation is maintained by METHYTRANSFERASE 1 (MET1), CHG by CHROMOMETHYLASE 2, 3 (CMT2 - CMT3), and DECRESED DNA METHYLATION 1 (DDM1), and CHH by CMT2 and DRM2 (Zhang et al., 2018; Gallego-Bartolome, 2020). If necessary, DNA methylation is erased by a mechanism called DNA demethylation.

DNA methylation occurs sparsely but throughout the genome, and thus exhibits ‘hot spots’. Methylation is observed in promoters, gene regions (especially gene body methylation) and transposable elements (TE). Promoters are *cis*-acting DNA domains that initiate gene transcription (Mithra et al., 2017). Methylation changes occurring in promoter regions usually suppress but exceptionally may even enhance gene expression (Zilberman et al., 2007). It has been proposed that DNA methylation may influence binding of either transcription activators or repressors, especially at promoters, and probably via condensation of the chromatin at the underlying locus (Brenet et al., 2011), although the exact mechanism remains poorly understood (Zhang et al., 2018). Furthermore, it has been suggested that promoter methylation can be a result of spreading of methylation from nearby transposons (Zhang et al., 2018). Gene body methylation usually occurs in the central part of genes away from transcription ends. In *A. thaliana* more than a third of genes are methylated (Zhang et al., 2006; Zilberman et al., 2007). The exact role of this phenomenon is not well understood but there are indications of a role in inhibition of transcription elongation, deactivation of cryptic transcription start sites and/or regulation of spurious transcription (Zhang et al., 2018; Le et al., 2020; Lucibelli et al., 2022). Finally, DNA methylation takes place also in TEs. TEs are mobile repetitive regions, that can move from one location to another in the genome, creating or deleting DNA sequences (Quesneville, 2020). They are divided into two classes (I and II), each of which includes different orders. Class I includes LTR retrotransposons (Gypsy and Copia), LINE (L1), and SINE (tRNA) whereas Class II contains TIR, with 8 different superfamilies, and HELITRON with only one (Quesneville, 2020). TEs are highly methylated and their methylation state regulates their mobility, the expression of nearby genes, and genome integrity (Zilberman et al., 2007; Ramakrishnan et al., 2021).

Both biotic and abiotic stresses can interfere with DNA methylation mechanisms and mostly (but not only) influence gene expression (Wang et al., 2011; Wang et al., 2019; Erdmann and Picard, 2020; Liu and He, 2020; Kryovrysanaki et al., 2022; Lucibelli et al., 2022). Especially during infections, DNA methylation has been found to control replication and infectivity of various viruses. For instance, during *Geminiviridae* infections, a group of DNA nuclear replicating viruses, DNA methylation induced by plant defense mechanisms controls geminivirus genome methylation levels, as well as plant TE mobility. Viruses counteract by initiating inhibition of global methylation through virus-encoded viral suppressor proteins (VSRs) (Wang et al., 2005; Raja et al., 2008; Coursey et al., 2018; Wang et al., 2019). The case of RNA viruses differs since no DNA replication step is involved. However, DNA methylation can occur in endogenous promoters, genes or TE affecting infection either positively or negatively. Pelargonium line pattern virus (PLPV) affects ribosomal DNA promoter upon infection through interference of methyltransferases and demethylases (Pérez-Cañamás et al., 2020). Studies in viruses, like tobacco rattle virus (TRV), cucumber green mottle mosaic virus (CGMMV), and cucumber mosaic virus (CMV) revealed changes in the infected plant methylome including in TE sequences, related to deregulation of global RdDM machinery (Wang et al., 2018; Diezma-Navas et al., 2019; Sun et al., 2019). Some, but not all, of these effects may occur through the action of the respective VSR. For example, CMV-2b, binds to AGO4, AGO6 and/or siRNAs, interfering with the RdDM pathway (Duan et al., 2012; Hamera et al., 2012). It is to note that another type of methylation (N6-methyladenosine - m6A) modification has been found in mRNA of all eukaryotes including viruses, influencing plant-virus interactions (Yue et al., 2022).

There have been several studies on the interaction between viral infections and DNA methylation up to this point, but there is limited information on viroid infections effects on DNA methylation. Viroids are small (246 to 401 nt) ncRNAs causing significant economic losses in crop plants (Flores et al., 2011; Katsarou et al., 2015; Katsarou et al., 2022). Their non-coding nature obliges them to use all possible aspects of their genome to induce infections, including affecting gene expression. It is known that DCLs target viroids (Papaefthimiou et al., 2001; Itaya et al., 2007; Dadami et al., 2013; Katsarou et al., 2016a; Kryovrysanaki et al., 2019) and produce vd-siRNAs of 21, 22 and 24 nt, that can be loaded onto different AGO proteins, including AGO4 and AGO6 (Minoia et al., 2014), potentially activating RdDM pathway. It has been shown that a viroid named potato spindle tuber viroid (PSTVd) can induce methylation of its own (Wassenegger et al., 1994; Wassenegger and Dalakouras, 2021) or of a foreign transgene (Lv et al., 2016). Furthermore, specific endogenous promoter regions of PSTVd-infected potato plants were shown to be hypermethylated, possibly with the implication of a known viroid partner, protein VirP1 (Kalantidis et al., 2007; Lv et al., 2016). In a recent study investigating genomic methylation levels in hop plants infected with various viroids, only hop latent viroid (HLVd) showed hypermethylation (Sečnik et al., 2022). On the contrary, in hop stunt viroid (HSVd) infections of either cucumber or *N. benthamiana* plants promoter regions of ribosomal genes and TEs were found hypomethylated through a direct interaction with HDA6 (HISTONE DEACETYLASE 6) protein, a known RdDM partner (Martinez et al., 2014; Castellano et al., 2015; Castellano et al., 2016a; Castellano et al., 2016b). In addition, during coinfections of PSTVd with a DNA virus (*Geminiviridae*) in tomato plants, hypermethylation of viral DNA was observed, whereas in coinfection of three viroids [citrus bark cracking viroid (CBCVd), HLVd and HSVd] a global genome hypomethylation was reported (Torchetti et al., 2016; Sečnik et al., 2022). All these studies show the degree of contradictory results concerning DNA methylation effect in viroid infections, and the need for supplementary studies to elucidate the phenomenon.

In this study, we investigated the interplay between PSTVd infection and DNA methylation in *N. benthamiana* plants. For this, we used a combination of chop-PCR, bisulfite sequencing, and MSAP-seq to study global methylation patterns upon viroid infection. We show that even though hypomethylation of specific targets is observed, the overall effect of DNA methylation during PSTVd infection remains marginal.

## 2 Materials and methods

### 2.1 Plant material and photographs

Wild-type *N. benthamiana* and DCL3.10i (Dadami et al., 2013; Katsarou et al., 2016a; Katsarou et al., 2018) plants were grown under stable greenhouse conditions. Nikon D5100 camera (Nikon, Tokyo, Japan) was used for plant photographs.

### 2.2 Bioinformatic analysis for detection of 24 nt population

We used previously published data from *N. benthamiana* plants infected with PSTVd for either 2 weeks post-infection (wpi) (MAC3/MAC4) (Di Serio et al., 2010) or 4wpi (MAC36/MAC37) (Navarro et al., 2021). 24-nt-long reads were selected and used for a bowtie2 (Langmead and Salzberg, 2012) mapping against the *N. benthamiana* available annotations (Niben.genome.v1.0.1) (Bombarely et al., 2012), including transposons and coding sequences (https://solgenomics.net/organism/Nicotiana_benthamiana/genome). GFF annotation file of *N. benthamiana* was used as a reference to extract the 1000 nt long upstream region of each annotated coding sequence (hereinafter referred to as promoters) using a custom Perl script. Previously isolated 24-nt small RNAs were aligned with no gaps or mismatches allowed by bowtie2 against the isolated promoter. Reads were normalized and log2-fold change was calculated to identify differentially expressed loci (**Table S1**).

### 2.3 Infections

*N. benthamiana* plants (WT and DCL3.10i) at the age of 4 to 6 leaves were infected with agroinfiltration as described before (Katsarou et al., 2018), using a construct containing PSTVd^NB^ – AJ634596 isolate kindly provided by Dr. De Alba and Dr. Flores (Institute for Cellular and Molecular Plant Biology—IBMCP). Samples for Chop-PCR, MSAP-seq, and bisulfite treatment were collected at 3 wpi. For the analysis of the 24 nt population *N. benthamiana* plants were infected mechanically (Di Serio et al., 2010) or by agroinfection (Navarro et al., 2021), and samples were collected at 2 wpi and 4 wpi.

### 2.4 RNA extraction and Northern blots

After collection and homogenization under liquid nitrogen, RNA extraction and Northern blots were performed as already described before (Katsarou et al., 2016a). Briefly, total RNA was extracted using Trizol reagent (38% saturated phenol -PanReac/Applichem, 0.8 M guanidine thiocyanate – Fisher Scientific, 0.4 M ammonium thiocyanate – Sigma/Aldrich, 0.1 M sodium acetate – Honeywell, 5% glycerol – Lach:ner), followed by phenol-chloroform treatment. RNA was precipitated using isopropanol (Honeywell) and salts (0.8M Na-citrate - PanReac/Applichem and 1.8M NaCl -Merck) and then washed with 70% ethanol (Scharlab). RNAs were diluted in 40μl H_2_O.

For Northern blot, five micrograms of total RNA were analyzed in 1.4% denaturating agarose gel (Invitrogen). RNAs were transferred in Nytran-N membrane 0.2μm (Whatman/GE Healthcare) via capillarity transfer followed by crosslink. PSTVd –DIG-labeled probe (DIG RNA labeling mix, Roche Diagnostics) was produced from plasmid *Hind*III-pHa106 (Tabler et al., 1992) using T7 polymerase (Takara).

### 2.5 DNA extraction

Total DNA was extracted using CTAB reagent (2% CTAB, 100 mM Tris pH 8, 20 mM EDTA, 1.4M NaCl, 1% PVP). The mix was incubated at 65°C for 20 min and was vortexed frequently. After chloroform cleaning, DNA was precipitated using isopropanol for 10 min at room temperature. The pellet was washed with 70% v/v ethanol (Scharlab) and diluted in 50 μl ddH_2_O with 0.2 mg/mL RNase A (QIAGEN) for 30 min followed by phenol/chloroform cleaning. Samples concentration was evaluated using Nanodrop ND-1000 spectrophotometer (Thermo Fisher Scientific).

### 2.6 Chop PCR

Ten ng/μl of genomic DNA from *N. benthamiana* wt and DCL3.10i plants both non-infected and PSTVd infected were incubated with 20U of *Mcr*BC, *Msp*I, and 25U of *Nla*III enzymes (cleaving at methylated (G/A)^m^C sites or unmethylated CG, CHC and CHH respectively) (all New England Biolabs). Three biological and at least three technical repeats were performed in each case unless mentioned differently in the text. *Mcr*BC and *Nla*III digestions were incubated overnight at 37°C, followed by enzyme inactivation at 65°C for 20 min. No star activity was noted for the enzymes used, as there was no nonspecific cleavage observed in the digestion of the unmethylated chloroplastic target. In the case of *Hpa*II, two technical replicates were incubated at 37°C overnight and inactivated at 80°C for 20 min. The third replicate was incubated at 37°C for two hours and then inactivated. No significant difference was observed between these two treatments. *MspI* digested samples were incubated for 2 hours at 37°C, but no inactivation was performed since *Msp*I is a non-heat-sensitive enzyme.

Following methylation-sensitive restriction enzymes (MSREs) digestions, PCRs were performed using 20 ng of DNA as template and Taq PCR (Minotech) according to the manufacturer’s instructions. Primers and annealing temperatures are described in **Table S2**. For proper quantification, we opted for 30 cycles during PCR. Amplicons were loaded in 1.5% agarose gels (Invitrogen) and photographed using Sapphire Biomolecular Imager (Azure Biosystems). Amplicon intensity was quantified with AzureSpot 2.0 software (Azure Biosystems). Each biological sample that was treated with an MSRE was compared with a non-digested control containing the same amount of gDNA treated in exactly the same way, but with direct inactivation of the enzyme or with no enzyme. The methylation status of each target was calculated by comparing the density of amplicons according to the following foa: $\left(MS=\frac{control-MSRE\;digestion}{\text{control}}\right)$ . The percentage of methylation for *Mcr*BC was calculated as MS*100, while for *Hpa*II, *Msp*I, and *Nla*III, it was calculated as (100-MS) *100 (Dasgupta and Chaudhuri, 2019).

### 2.7 MSAP-seq

Five hundred nanograms of genomic DNA was digested with 30U of the rare-cutter restriction enzyme *EcoR*I-HF (New England Biolabs) and 20U of the frequent-cutter methylation sensitive restriction enzyme *Hpa*II (New England Biolabs) in a final volume of 50 μl. The reaction was incubated overnight at 37°C, followed by inactivation at 80°C for 20 min. Then, phenol/chloroform and ethanol precipitation was performed with a final DNA concentration of 15 ng/μl. *EcoR*I and *Hpa*II adapters [(Chwialkowska et al., 2017) - **Table S2**] were hybridized (95°C for 5 min) and then cooled down at RT. The ligation reaction (20μl) contained 216 ng of digested DNA, 6 pmol of *EcoR*I and 60 pmol of *Hpa*II pre-hybridized adapters and was performed according to manufacturer instructions at 16°C (New England Biolabs). Then 50 μl of PCR was set (5μl 10x Taq DNA polymerase buffer, 25 mM MgCl_2_, 1.5U Taq DNA pol, 0.32 mM of each deoxynucleotide triphosphate) as proposed by the manufacturer (Minotech) using 33 ng of the ligated DNA and 0.32 μΜ of A1 strand of each adapter (EcoRI_A1 and HpaII_A1). PCR conditions were the following: 30 cycles of 94°C for 30 sec, 56°C for 40 sec, 72°C for 75 sec. We performed four independent replications for WT and PSTVd infected plants that were purified using 1.8x Agencourt AMPure XP (Beckman Coulter) and eluted into 50 µL 1x TE. Following, sonication using 10 cycles of 30 sec with 30 sec intervals was performed in a S220 Ultrasonicator (Covaris) creating fragments of around 300 bp which were again purified using 1.8x Agencourt AMPure XP and eluted into 35 µL ddH_2_O. The purified fragments were analyzed in a bioanalyzer 2100 (Agilent). Construction of libraries and High throughput sequencing (HTS) were performed at the IMBB-FoRTH sequencing facility.

### 2.8 MSAP-Seq bioinformatic analysis

The quality of the raw reads was evaluated using FastQC (Andrews, 2010). The preparation of the data followed the MSAP-Seq protocol as described in (Chwialkowska et al., 2017). More specifically, reads containing a modified *Hpa*II adapter sequence (GACGATGAGTCTAGAA) were retained using seqkit grep (Shen et al., 2016). Adapters were then trimmed using BBDuk (Bushnell, n.d), with the following parameters: k=16, ktrim=l, qtrim=r, trimq=20, minlen=50, keeping reads with a minimum length of 50 bp. Reads were then filtered based on the presence of the CGG sequence, either on the 5’ or 3’ end. Reads were then aligned to the *N. benthamiana* v1.0.1 (Bombarely et al., 2012) reference genome obtained from Sol Genomics (https://solgenomics.net), using bowtie2 (Langmead and Salzberg, 2012) with settings: –end-to-end, –n-ceil L,0,0.05. Indexed and sorted bam files were produced with Samtools (Danecek et al., 2021) and converted to the bed format using the bedtools suite (Quinlan and Hall, 2010), only retaining aligned reads mapping to a chromosome region with a CCGG tetranucleotide. Using the msgbsR package (Mayne et al., 2018), CG positions were counted and normalized by CPM (counts per million), with loci having a minimum coverage of 1 CPM in at least 3 samples of either condition origin being retained for downstream analysis. Statistical analysis was performed using an edgeR (Robinson et al., 2009) wrapper provided by the msgbsR package. Positions having a p-adjusted value ≤ 0.05 were deemed as DMPs (Differentially Methylated Positions) and any functional elements they colocalized with as DMRs (Differentially Methylated Regions). CG positions were annotated using information from Sol Genomics (https://solgenomics.net) and categorized into: genes [from transcription start site (TSS) to the end, including exons and introns], putative promoters (1,000 bp upstream of the TSS), transposable elements [TEs, as classified in the Dfam database (Storer et al., 2021)], other repeat regions (non TEs) and intergenic regions (un-annotated). Principal component analysis (PCA) was carried out using the PCAtools (Blighe and Lun, 2020). The ComplexHeatmap (Gu et al., 2016) R package was used for hierarchical clustering and heatmap construction.

### 2.9 Bisulfite treatment

Bisulfite conversion was performed using the EpiTect bisulfite kit (QIAGEN) on biological samples of non-infected and PSTVd-infected *N. benthamiana* plants. One microgram of genomic DNA was treated according to the manufacturer’s instructions. Modified DNA was then amplified by PCR using Taq polymerase (MINOtech), with primer sequences and conditions described in **Table S2**. The amplified DNA was cloned into T-tailed cloning vectors (pBluescript SK(-) vector or pGEM-T Easy vector - Promega). As control for conversion efficiency, clones of chloroplastic DNA were Sanger sequenced, and after verification of a full conversion, the rest of the samples were sequenced. All obtained sequencing results were aligned using Mega 11 software with Muscle (Tamura et al., 2021). Three different types of analyses were performed. a) For cellular synthase and germin-like promoters, Kismet web software was used (Gruntman et al., 2008). Mann-Whitney with non-parametric variables was used for the statistical analysis. b) For the TE, methylation analysis was performed by eye to avoid confusion with possible mutations, since TEs are found in different places in the genome. c) For Aquaporine-4, MATE, and PLATZ, we identified the CCGG region of the sequenced amplicon that corresponds to the DMP identified in the MSAP-Seq analysis. Four biological samples were sequenced (the same used for MSAP-Seq experiments). The percentage of ^5m^C was calculated by dividing the number of methylated cytosines by the total number of clones in each testing sample.

### 2.10 Software

For verification of promoters regions of targets, we used PlantCare (https://bioinformatics.psb.ugent.be/webtools/plantcare/html/) (Lescot et al., 2002). Figures were created using Adobe Photoshop 2020 (Adobe Systems Inc.), and Adobe Illustrator 2020 (Adobe Systems Inc.). Graphs were created using GraphPad Prism 8 software (GraphPad Software Inc, 2021). For GO analysis PlantRegMap was used (http://plantregmap.gao-lab.org) (Tian et al., 2020). Information about the used pipeline is available on https://github.com/GCMLab-Forth/MSAP-seq_analysis.

## 3 Results

### 3.1 *N. benthamiana* promoters and TE affected by 24nt siRNAs during PSTVd infection

**Figure 1:**
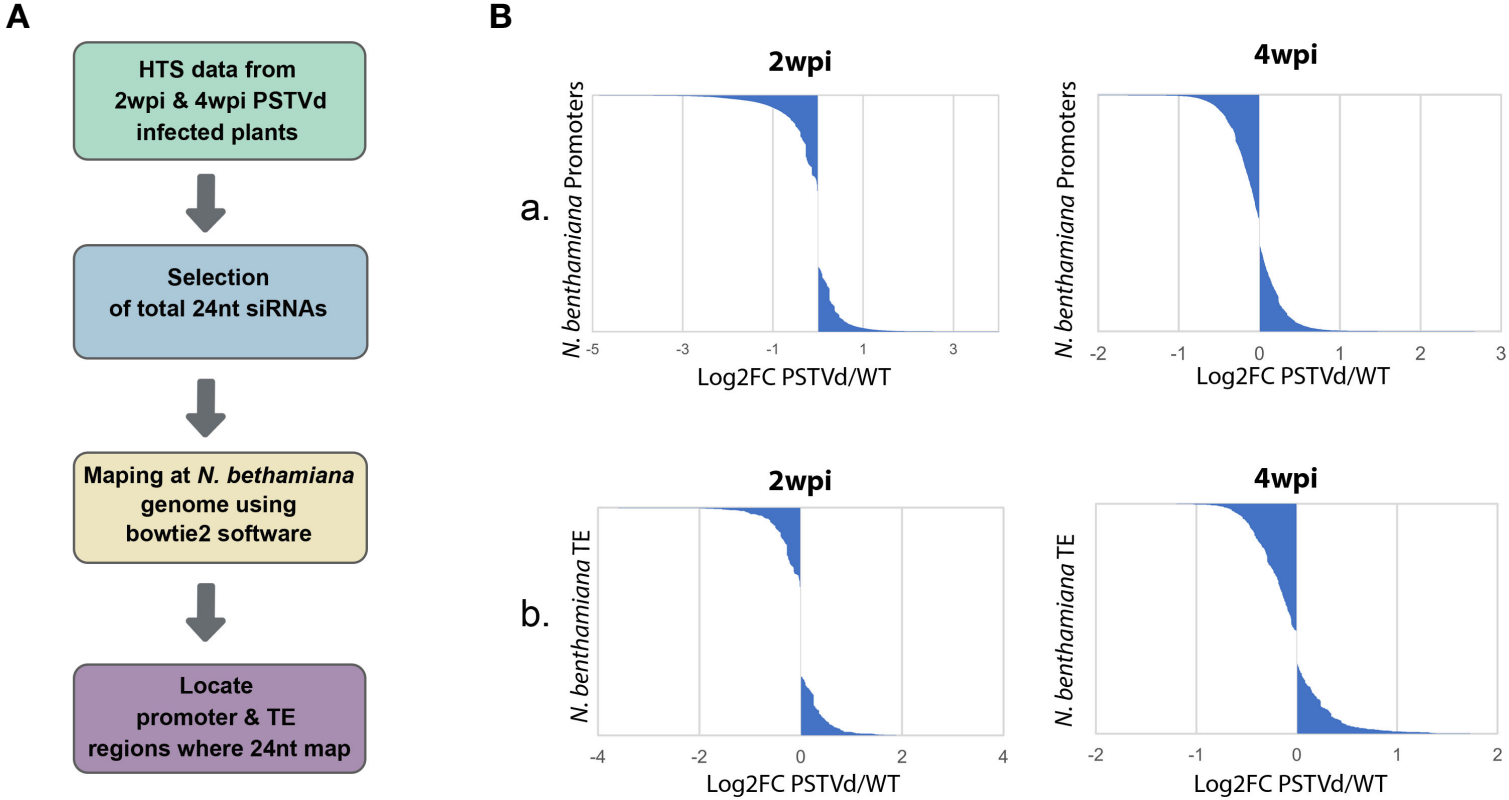
Mapping of the 24nt siRNAs population from non-infected and PSTVd infected N. benthamiana plants in the promoter and TE regions. **(A)** Schematic representation of the used bioinformatic pipeline **(B)** Graphical representations of the mapped 24 nt siRNAs of WT versus PSTVd-infected plants in promoter region **(A)** and TE **(B)** at 2 wpi and 4 wpi (details in **Table S1**).

As previously mentioned, CHH methylation is mainly driven by the presence of 24 nt siRNAs (Erdmann and Picard, 2020). Therefore, we decided to investigate the possibility that 24 nt produced both in WT and PSTVd-infected plants can target promoters and TE which will then result in alterations in DNA methylation. To this end, we used previously published HTS data of WT PSTVd-infected vs. not-infected at two time points, 2 wpi (Di Serio et al., 2010) or 4 wpi (Navarro et al., 2021). We used two different time points, since siRNAs may change in number and type during infections as has been previously shown for a different viroid (Márquez‐Molins et al., 2023). We extracted the 24 nt siRNA population of these samples and mapped them using both a region of 1,000 nt upstream of the initiation site of each gene and the identified TE of *N. benthamiana* expected in plants to contain the promoter region (details in material and methods). It is to be noted that typically in plants, as promoter region we consider a 500 to 1,000 nt region upstream of the transcription initiation site (Mithra et al., 2017). As shown in **Figure 1** and **Table S1**, we did not observe significant differences in terms of absolute population size between the 24 nt siRNAs mapping into promoters and TE between WT and PSTVd infected plants. It is to be noted that libraries produced at 2 wpi had five times less depth compared to 4 wpi, resulting in a lower number of identified (promoters and TEs).

### 3.2 Methylation status of specific promoters and TE in PSTVd-infected plants

Upon further investigation of the above bioinformatic analysis, we observed a few promoters and TE where the 24 nt are mapped in higher or lower numbers in the PSTVd-infected plants compared to the non-infected ones. To test methylation levels of the DNA at these specific loci, our method of choice was chop-PCR, a technique where methylation-sensitive restriction enzymes (MSREs) are used on DNA, followed by PCR amplification (Dasgupta and Chaudhuri, 2019). We opted for PCR and not qPCR firstly because it is a cost-effective procedure and secondly to increase the number of potential targeted regions by the MSRE in the analyzed sequence. We started by identifying possible targets. We selected sequences that were identified in both available *N. benthamiana* databases (https://solgenomics.net, and https://www.benthgenome.qut.edu.au/), excluding non-identified nucleotides (N) in their sequence, and with significant reads numbers. In addition, for the selection of promoter regions, we confirmed the presence of typical promoter motifs such as TATA and also confirmed expression variation in publicly available datasets (Owens et al., 2012; Katsarou et al., 2016a; Katsarou et al., 2016b; Xia et al., 2017). For TE, we preferred sequences that were present in different regions, as a TE is expected to be. In total from the above data, we have identified a germin-like promoter and an LTR/Copia type TE as possible targets to further analyze (**Table S3**). GERMIN-LIKE genes are involved in disease resistance in various crops (Dunwell et al., 2008) and are found with altered expression in published data sets (data not shown). Additionally, as positive control, we used the cellulose synthase *N. benthamiana* promoter which has previously been shown to be methylated in potato (Lv et al., 2016) (**Table S3**).

**Figure 2:**
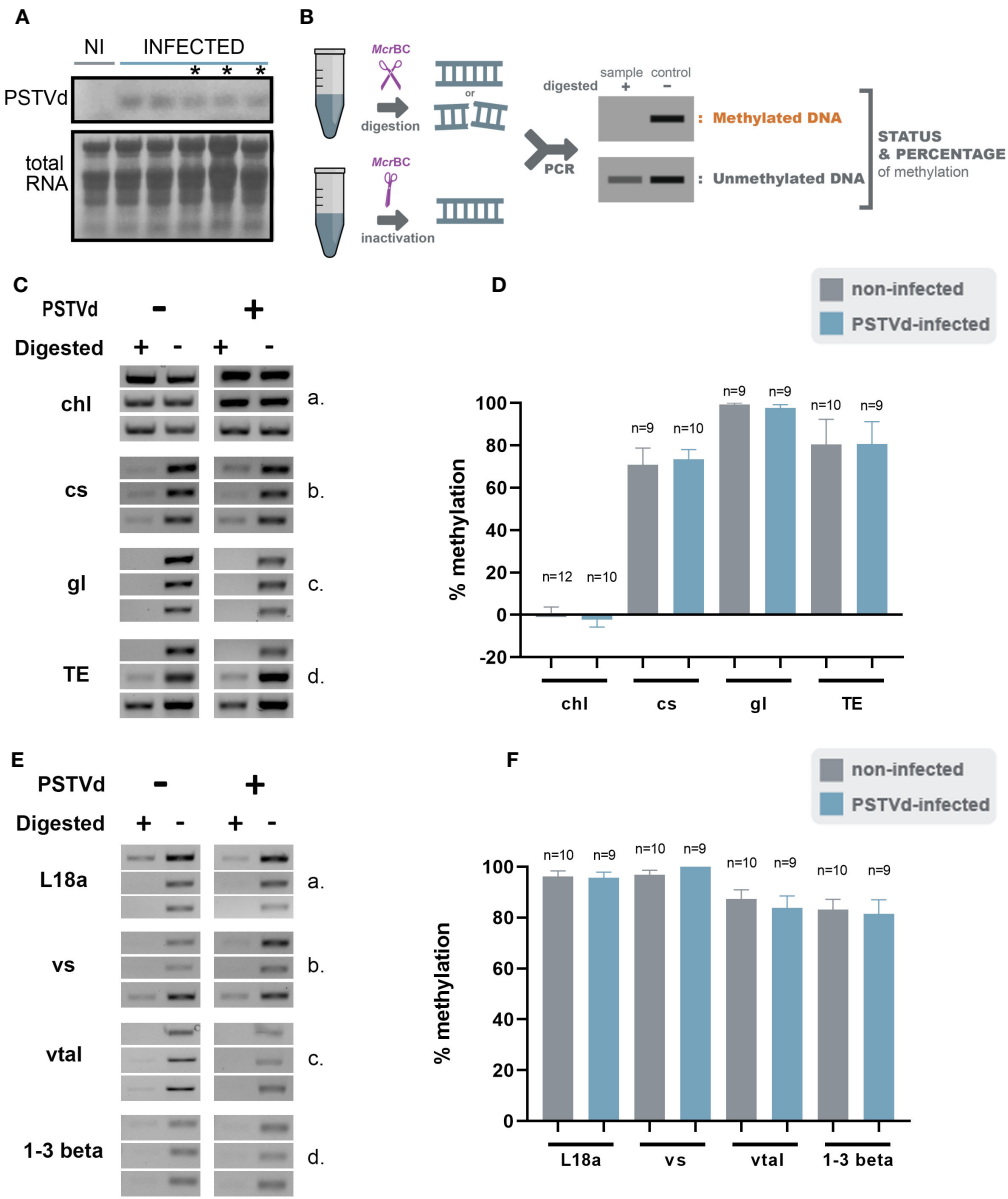
*Mcr*Bc digestions in non-infected and PSTVd-infected plants. **(A)** Northern blot analysis of WT and PSTVd-infected *N. benthamiana* plants (3 wpi). Total RNA identified with methylene blue was used as a loading control. (*) stands for samples used for further analysis. **(B)** Schematic representation of the *Mcr*BC principle. **(C, E)** Chop PCRs using *Mrc*BC in DNA of WT and PSTVd-infected plants. Extracted DNA was cut with *Mcr*BC followed by PCR amplification. If methylated no band is observed in the digested sample. Enzyme inactivation was used as control. Used targets were a control DNA (chl - C,a), promoter region of cellulose synthase (cs - C,b), germin-like (gl – C,c), TE Cc), ribosomal L18a (E,a), vironine synthase (vs - E,b), vacuolar protein sorting - associated protein (vtaI - E,c) and glucan endo 1-3 beta – glucosidase (E,d). Triplicates were made for each digestion. One is presented here and two more in **Figure S2** **(D, F)** Graphical representation of chop PCRs. ‘n’ represents the number of calculated replicates from all biological and technical repeats.

We then infected *N. benthamiana* plants with PSTVd and at 3 wpi plants had the characteristic phenotype with plant stunting and slight yellowing of the leaves (**Figure S1**). Northern blot analysis was performed to verify infection (**Figure 2A**). Samples with similar infection levels were selected for further analysis.

To determine DNA methylation status of the selected targets, we created specific primers (**Table S2**) and performed chop-PCR. We started the analysis using *Mcr*BC, an MSRE that cleaved methylated DNA with two half-sites of the sequence (G/A)^m^ C (Dasgupta and Chaudhuri, 2019). If this motif is present/methylated in the target region, no PCR amplicon is produced whereas, amplicons are produced in non-methylated regions or untreated samples (**Figure 2B**). Three biological and at least three technical repeats were performed for statistical reasons. Used primers are presented in **Table S2** and quantifications of the bands were performed as described before (Dasgupta and Chaudhuri, 2019) (see also material and methods). We have tested the methylation status of cellulose synthase promoter, germin-like promoter, and the LTR/Copia TE in WT and PSTVd-infected plants. A methylation-insensitive internal DNA control (chloroplastic DNA) was used (Ahlert et al., 2009). As shown in **Figures 2C, D**, and **S2** no difference in DNA methylation levels was observed upon PSTVd infection.

We questioned whether this was due to improper target selection. Therefore, we identified a few additional targets using the same guidelines and treated DNA as before (**Table S3**). We selected the promoter of the ribosomal protein L18a, a protein involved in virus infections (Li et al., 2018), the promoter of vironine synthase an acetyltransferase participating in alkaloid biosynthesis (Pfitzner et al., 1986), the promoter of vacuolar protein sorting - associated protein 1 a protein influencing the multivesicular bodies (MVB) pathway (Williams and Urbé, 2007) and finally the promoter of glucan endo 1-3 beta – glucosidase, an enzyme stimulated upon fungal, bacterial and pathogens infections (Kebede and Kebede, 2021). Results are presented in **Figures 2E, F**, and **S3**, showing that no difference was observed in methylation levels. It is noted that the positive control target, cellulose synthase, which was previously shown to be methylated upon viroid infection in potato (Lv et al., 2016), did not exhibit the same effect under our experimental conditions.

**Figure 3:**
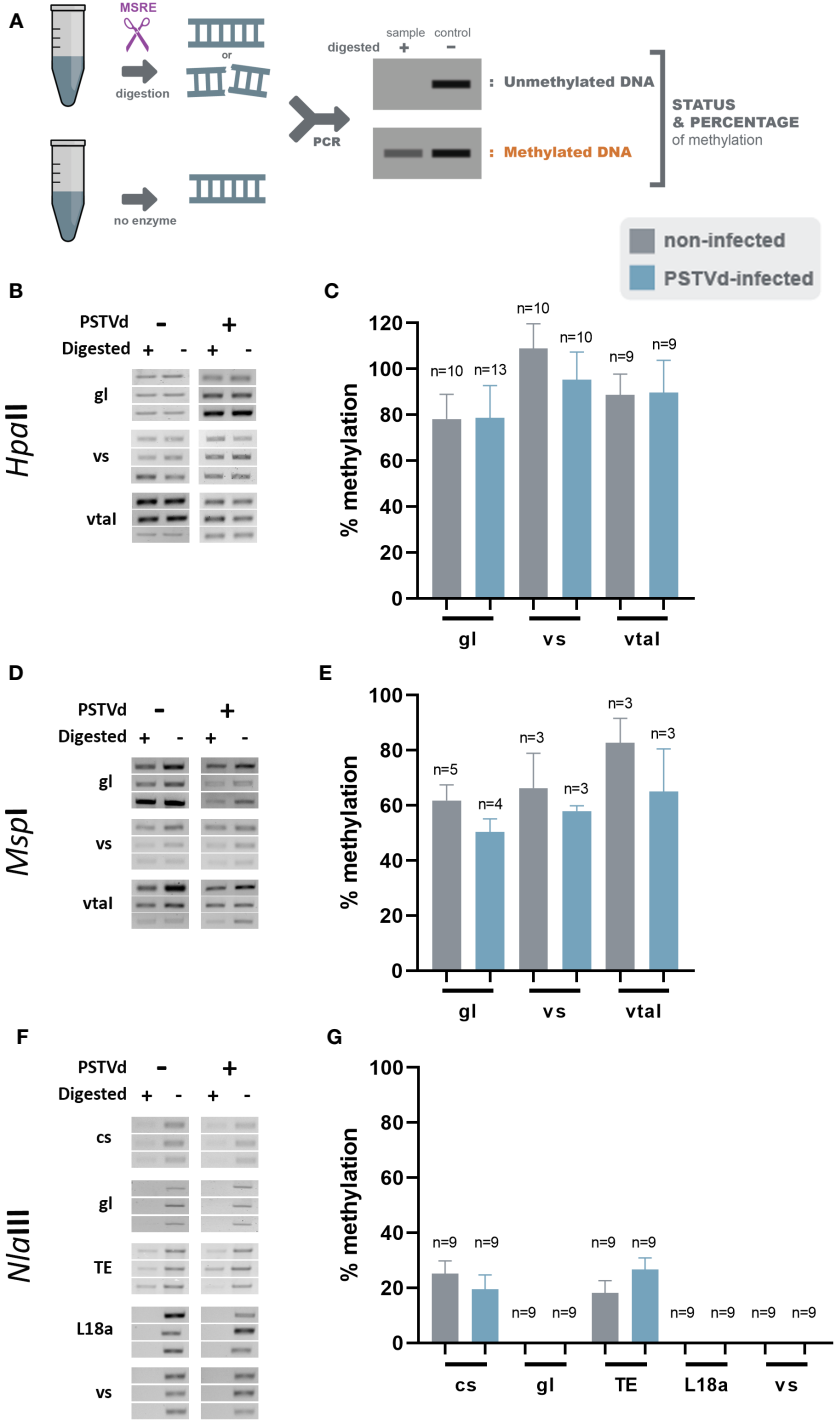
*Hpa*II, *Msp*I and *Nla*III digestions in non-infected and PSTVd-infected plants. **(A)** Schematic representation of the digestion pattern and result obtain during MRSE digestions. All used enzymes cut unmethylated DNA, therefore a band is expected after PCR if methylation occurs. Three biological and three technical repeats were performed (one presented here and two in **Figure S3**) using *Hpa*II **(B)**, *Msp*I **(D)** and *Nla*III **(F)**, for promoter region of germin-like (gl), vironine synthase (vs), vacuolar protein sorting - associated protein (vtaI), cellulose synthase (cs), ribosomal L18a (L18a), glucan endo 1-3 beta – glucosidase (1-3 beta).and TE. **(C, E, G)** Graphical representation of chop PCRs measurements, respectively. ‘n’ represents the number of calculated replicates from all biological and technical repeats.

Since small changes in specific nucleotide patterns can be underestimated when using the *Mcr*BC enzyme, we investigated changes in each pattern of methylation separately. Specifically, we used methylation sensitive *Hpa*II, *Msp*I, and *Nla*III restriction enzymes for analysis of CG, CHG, and CHH contexts, respectively (**Figure 3A**). Chop-PCRs, in selected targets containing a pattern recognized by each MSRE, identified no differences in the methylation of the specific sites between WT and PSTVd-infected plants for all tested targets (**Figures 3**, **S2**). It should be noted that only one replicate with three biological repetitions was performed in the case of *Msp*I, but the results were the same.

**Figure 4:**
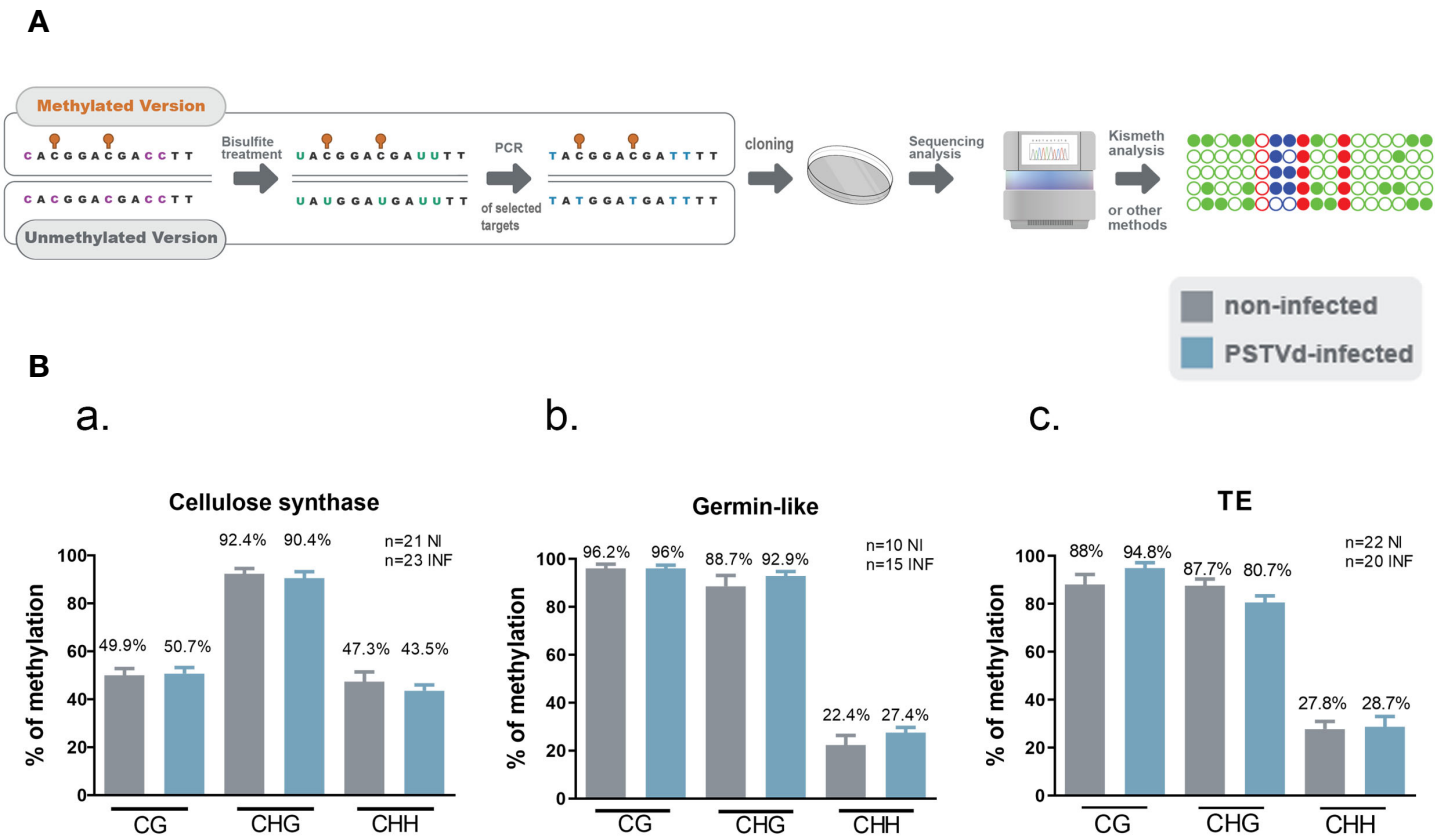
Bisulfite Sequencing for targets cellulose synthase, germin-like and TE in non-infected and PSTVd infected samples. **(A)** Schematic representation of the Bisulfite sequencing conversion and experimentation. **(B)** Graphical representation of Bisulfite sequencing results from cellulose synthase (a), germin-like (b) and TE (c) targets. Results are presented with SE. Statistical analysis was performed using Mann-Whitney test with non-parametric variants, without any significance being identified. ‘n’ stands for the number of clones sequenced in each case. NI for non-infected and INF for PSTVd-infected.

Given that no differences were observed in any of the selected targets with any of the tested MSRE, we questioned whether our Chop-PCR lacked sensitivity for this analysis where the differences in methylation levels may be too subtle to be revealed by this semi-quantitative approach. Hence, we performed Bisulfite Sequencing (BS) experiments for germin-like promoter, cellulose synthase, and LTR/Copia TE targets. This technique involves treating DNA with bisulfite to deaminate unmethylated cytosines and convert them to uracils, without affecting methylated cytosines (**Figure 4A**). We used chloroplastic sequences as a control of the conversion. We sequenced more than 10 clones in each case, as shown in **Figure 4B**, and no differences were observed in CG, CHC, or CHH contexts upon PSTVd infection. Taken together, results from both Chop-PCRs and BS consistently showed no changes in the DNA methylation status of the selected targets upon PSTVd infection.

### 3.3 The 24 nt siRNAs are probably not involved in DNA methylation changes upon PSTVd infection

**Figure 5:**
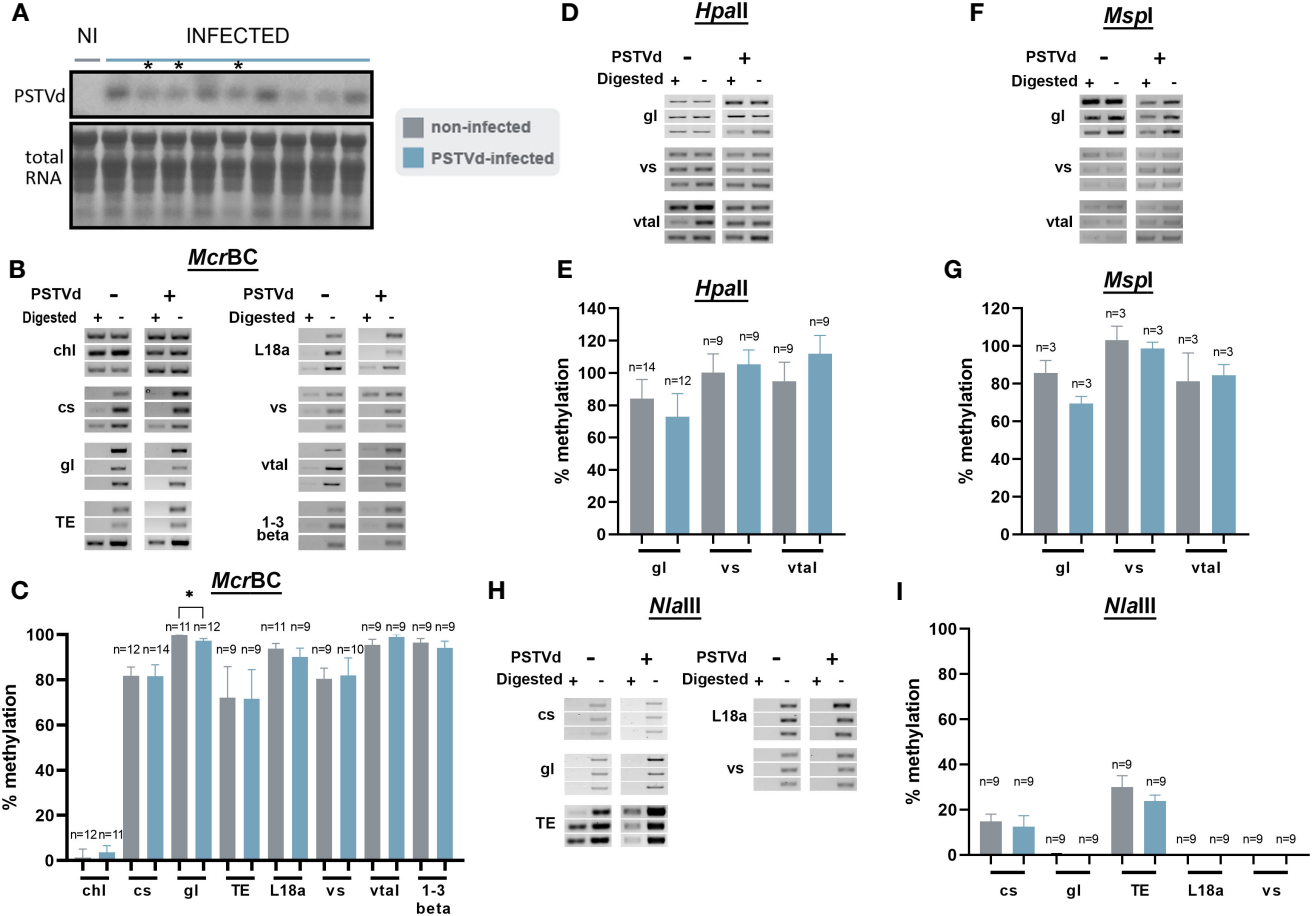
Chop-PCR in non-infected and PSTVd-infected DCL3.10i *N. benthamiana* plants. **(A)** Northern blot analysis of DCL3.10i and PSTVd infected *N. benthamiana* plants (3wpi). Total RNA identified with methylene blue was used as a loading control. (*) Symbolizes the infected plants that were selected. Digestion of DNA from DCL3.10i non-infected and PSTVd infected plants with *Mcr*BC **(B)**, *Hpa*II **(D)**, *Msp*I **(F)**, *Nla*III **(H)**, in triplicates of one biological repeat. Two more are present in **Figure S4**. Used targets were a control DNA (chl), promoter region of cellulose synthase (cs), germin-like (gl), TE, ribosomal L18a (L18a), vironine synthase (vs), vacuolar protein sorting - associated protein (vtaI) and glucan endo 1-3 beta – glucosidase (1-3 beta). **(C, E, G)** and **(I)** Graphical representation of chop PCRs measurements. ‘n’ represents the number of analyzed samples from all biological and technical repeats. Results were analyzed with unpaired Student t-test, with level of significance set at p< 0.05 and symbolized with (*).

Since no DNA methylation variations were observed in PSTVd-infected *N. benthamiana* plants for the loci we tested above, we considered the possibility that methylation changes are masked by the overall high level of methylation especially in the case of TEs. Therefore, we opted for *N. benthamiana* DCL3 knock-down plants (DCL3.10i) shown to have a decrease in the number of 24 nt reads (Katsarou et al., 2016a; Katsarou et al., 2018). As mentioned before the 24 nt population has been involved in methylation through the RdDM pathway (Erdmann and Picard, 2020) and *A. thaliana* dcl3 knock-out plants have been shown with reduced methylation status (Xie et al., 2004; Henderson et al., 2006; Stroud et al., 2013). Infections were performed and northern analysis verified that plants were infected (**Figures 5A**, **S1**). Chop-PCRs were performed using *Mcr*BC, *Hpa*II, *Msp*I, and *Nla*III in three biological and at least three technical repeats as before (**Figures 5**, **S4**). As shown, apart from germin-like promoter using *Mcr*BC, no obvious differences were observed with the use of all MSREs, suggesting that even in DCL-suppressed background, no significant methylation differences were observed upon viroid infection.

### 3.4 Investigate the methylation status of endogenous targets

**Figure 6:**
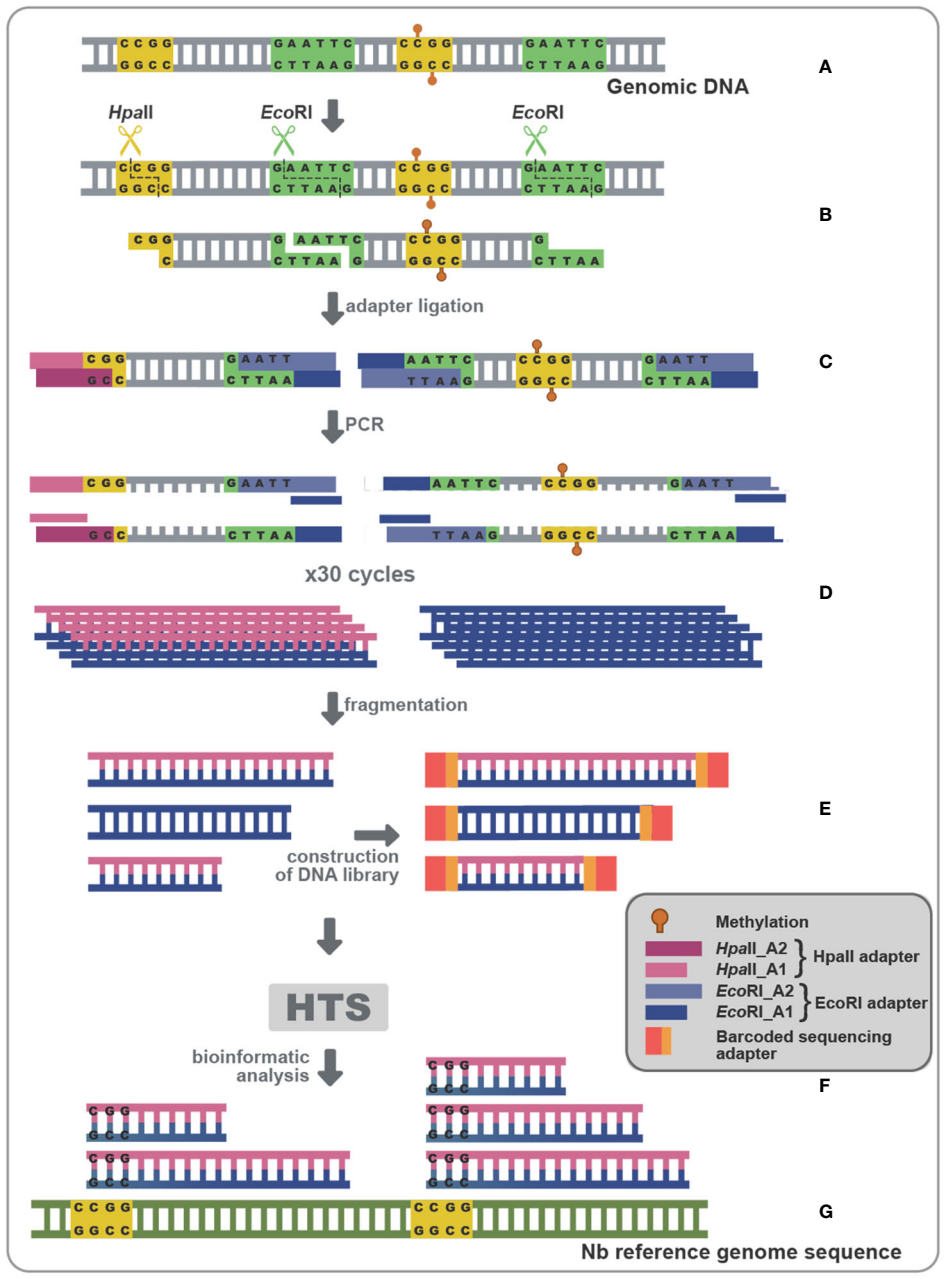
MSAP-Seq. Schematic representation of the MSAP-Seq pipeline. After genomic extraction with an appropriate protocol **(A)**, *Eco*RI, a rare cutter and *Hpa*II, a MRSE recognizing unmethylated cytosines in the pattern CCGG were used **(B)**, followed by ligation of adapters (*Hpa*II-A1/*Hpa*II-A2 and *Eco*RI-A1/*Eco*RI-A2) containing a known region **(C)**, which allowed for a PCR amplification **(D)**. Then, a sonication step drives fragmentation of PCR parts, followed by a library construction **(E)** and a High-throughput sequencing (HTS) **(F)**. Finally, results are analyzed using a bioinformatic pipeline **(G)**.

Since the use of Chop-PCR and BS on specifically chosen targets did not show significant methylation status alteration, we reasoned that this could possibly result from suboptimal target selection. In an attempt to overcome such a potential limitation, we opted for a technique that could give us a more global idea of the methylation status during viroid infection (Chwialkowska et al., 2017). Methylation Sensitive Amplification Polymorphism Sequencing (MSAP-Seq) is a variation of the classical MSAP technique used for a long time for methylation-related studies (Reyna-López et al., 1997; Xiong et al., 1999; Peraza-Echeverria et al., 2001; Salmon et al., 2008; Wang et al., 2011). As schematized in **Figure 6A**, with this technique DNA is digested with both *Eco*RI, a rare cutter that recognizes the GATTC site, and an MSRE, in this case *Hpa*II which recognizes and cuts unmethylated CCGG sites. Then the *Eco*RI-*Hpa*II fragments are ligated with specific *Eco*RI and *Hpa*II compatible dsDNA adaptors (**Table S2**) and amplified using nonselective primers complementary to the adaptors (MSAP_*Eco*RI_A1 and MSAP_*Hpa*II_A1-**Table S2**). Finally, bioinformatic analysis is performed, to identify Methylated Positions (MPs) that contain a CG site at either the 5’ or 3’ end of each read, followed by quantification of the Differentially Methylated Positions (DMPs) between non-infected and PSTVd infected samples (**Figure 6**). Details about the reads of each library as well as reads during the bioinformatic analysis are presented in **Table S4**. We performed MSAP-Seq on samples from four non-infected plants and four PSTVd-infected plants. Initially, we investigated the potential of the technique. To this end, we measured the number of consensus sequences theoretically present in *N. benthamiana* genome. In total, 1,534,792 sites were identified, whereas, during the actual experiment 137.192 (9%) sites were found. This suggests that either there is a high methylation (100% methylation at both alleles of a given locus) abundance throughout the *N. benthamiana* genome or that there is a limitation in this technique probably related to the specific enzymes used. Regardless of this technical interrogation, we determined 240 sites as DMPs between healthy and infected plants implying that overall, only a few targets are showing differences in ^5m^C methylation levels at the CG context upon PSTVd infection.

**Figure 7:**
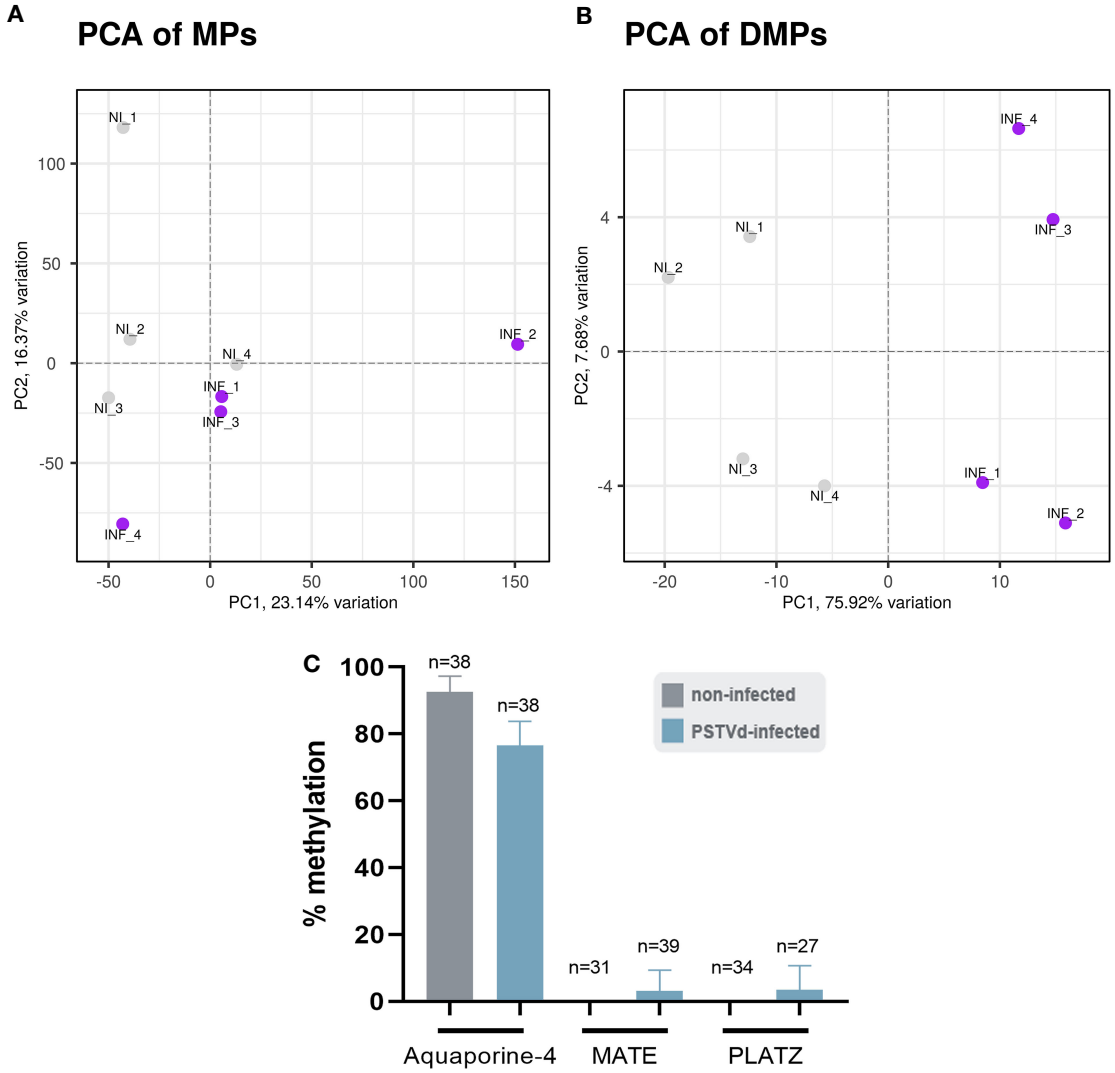
Principal component analysis (PCA). A difference in the clustering of analyzed samples was observed between non-infected (NI) and PSTVd infected plants (INF) as shown by **(A)** PCA for Methylated Positions (MP) and **(B)** PCA for Differentially Methylated Positions (DMP). **(C)** Bisulfite sequencing analysis for bioinformatic pipeline verification. Three promoter targets were chosen, Aquaporine-4, MATE and PLATZ. ‘n’ represents the number of sequenced clones analyzed.

We performed the bioinformatic analysis using the recently published msgbsR package (Mayne et al., 2018) which was released during the course of our study and which reinforced statistical power over preliminary results we generated (data not shown) with scripts derived from previously published MSAP-seq analysis (Chwialkowska et al., 2017). First, we operated a principal component analysis (PCA) to represent MPs and DMPs obtained for each replicate. As shown in **Figure 7A**, a slightly different clustering was observed in MPs between non-infected (NI) and PSTVd-infected (INF) plants with a percentage of variance between them (PC1) of 23.14%. This suggested a small yet measurable difference in methylation level between samples, which was further pronounced when only DMPs were analyzed and where PC1 identified 75.92% of variance (**Figure 7B**).

To verify that the analysis was reliable, we performed BS for three promoter regions. We chose promoters of Aquaporine-4, Multi antimicrobial extrusion protein (MATE) efflux family protein and plant AT-rich protein, and zinc-binding protein (PLATZ) transcription factor (details in **Table S3**), all of which presented a high number of reads at a CG position and significant LOG_2_FC alteration (**Figure S5**; **Table S5**). The primers used for BS can be found in **Table S2**. For each condition, we used the same four biological samples used for the MSAP-Seq, with at least 10 clones sequenced *per* case. As shown in **Figure 7C**, Aquaporine-4 target showed a tendency for hypomethylation as was expected whereas no alteration was observed in the other two promoters. This discrepancy could be due to the stochastic nature of this technique, since it is known that BS is highly dependent on the number of clones sent for sequencing (Darst et al., 2010). Therefore, we considered our bioinformatic analysis valid.

**Figure 8:**
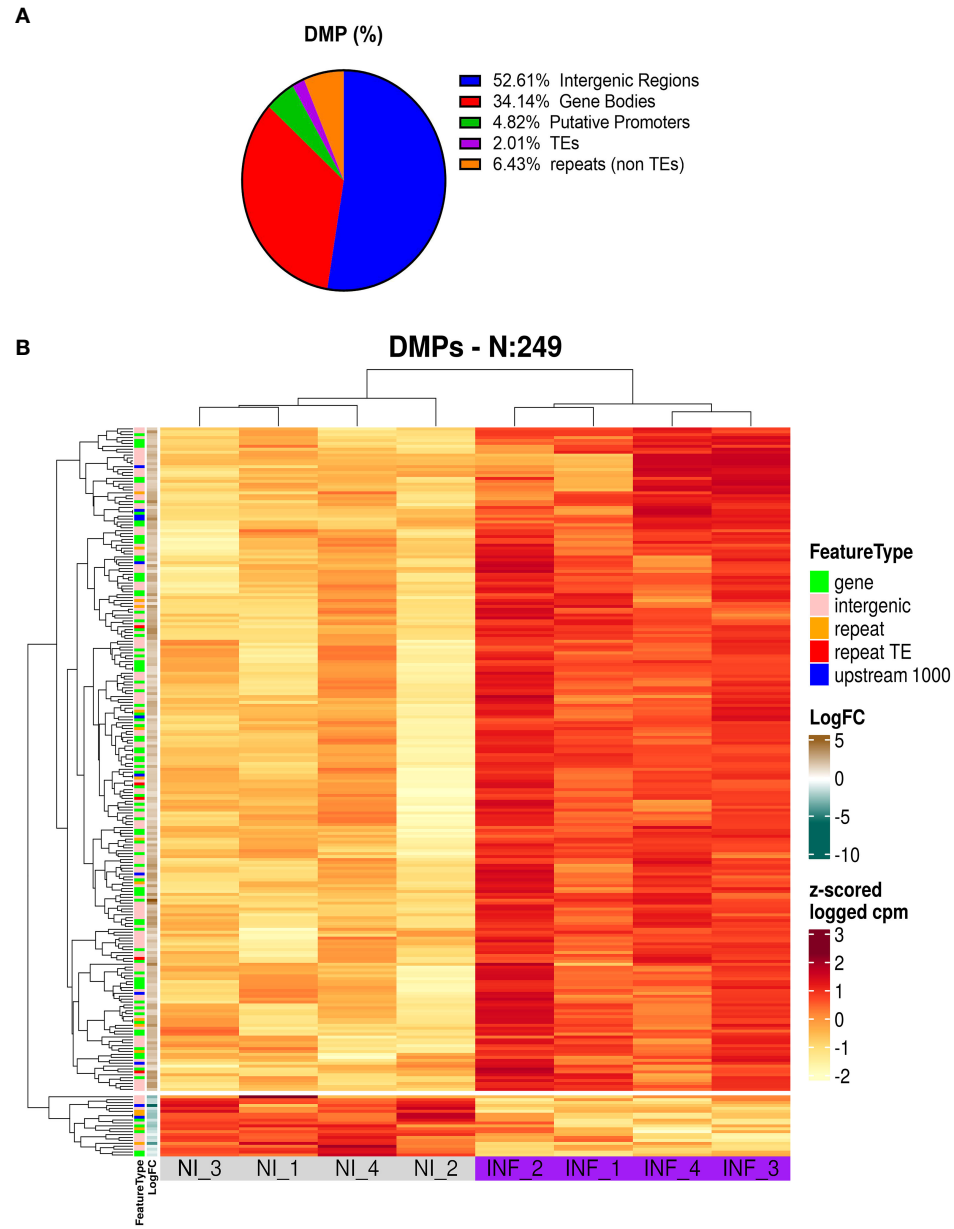
Results of MSAP-Seq. **(A)** Analysis of DMP localization in *N. benthamiana* genome. **(B)** Heatmap of DMP found in PSTVd infected plants.

To identify the genomic distribution of the changes in DNA methylation, and check if some particular cellular function maybe affected, we analyzed DMPs in putative promoters’ regions (1000 nt upstream of the transcription site), gene body, intergenic regions, TEs, and repetitive regions. As presented in **Figure 8A** and **Table S4** most of the DMPs (52.6%) are found in intergenic regions. This is expected given the proportion of the genome spanned by these regions. However, we found an enrichment in gene body areas (34.1%), and putative promoters (4.8%). TEs and repeats (other than TEs) have the lower number of DMPs with 2 and 6.4% respectively. It is important to note that we obtained 9 DMPs that were identified in more than one location (e.g. overlapping Promoter and TE).

Next, we performed a heatmap to identify differences in all tested samples. As mentioned before, *Hpa*II cuts only sites with unmethylated cytosines, therefore an increase in the number of the reads corresponds to hypomethylation and *vice versa* a decrease in the number of reads translates into a hypermethylation event. As shown in **Figure 8B** and **Table S5**, our data suggests that most of the DMP show hypomethylation and only 21 DMPs are hypermethylated. We next attempted functional analysis by considering the DNA methylation alterations in promoters, gene bodies, or TEs. Considering the gene names of hits in gene bodies and their associated ontology, we identified an enrichment in GO terms that are related to microtubules movement/activity, movement of cell or subcellular component (e.g Niben101Scf03403g03041.1 - ATP binding microtubule motor family protein) as well as proteins related to regulation of reproductive processes (e.g Niben101Scf00320g01005.1 - MADS-box transcription factor 3) (**Figure S6A**; **Table S6**). Then we focused on promoters that had at least one position differentially methylated. We identified 12 promoters, which after GO enrichment of the respective proteins, were mostly involved in transport (e.g. Niben101Scf20773g01021.1 – Aquaporine-4) (**Figure S5B**; **Table S6**). In addition, the promoter of a bromoprotein, was found with a CG hypomethylated during PSTVd infection. Sequence comparison with bromoproteins of *A. thaliana* and tomato (data not shown) identified this protein as GTE2-like protein, a probable transcription factor probably related to reduced success of agrobacterium-induced root transformation (Ma Crane and Gelvin, 2007). Finally, we investigated TE methylation and identified only 5 of them being regulated during infection (**Table S5**), which might seem low but in general TEs are heavily methylated. Finally, we compared the results presented here with microarray experiments previously published by our laboratory (Katsarou et al., 2016a). At that time, we had infected *N. benthamiana* plants with PSTVd and after four weeks analyzed transcriptome expression. As shown in **Table 1**, we found two promoter regions (promoter of E3 ubiquitin-protein ligase TRIM39 and Receptor-like protein Kinase) that had shown altered expression during infection and were also identified in MSAP-Seq analysis of this work, suggesting a possible correlation between methylation and transcription levels. Additionally, when we looked at previously published data from PSTVd infected *N. benthamiana* (Katsarou et al., 2016a; Katsarou et al., 2022), we found no changes in the levels of transcripts and proteins involved in the RdDM pathways. Taken together, our results suggest mild changes in the overall CG methylation status of *N. benthamiana*. However, specific targets in promoter regions, TEs and gene body regions were found to be altered significantly and might need further investigation to determine to what extent they drive viroid effects in *N. benthamiana.*

**Table 1:** Comparison of results obtained with MSAP-Seq and previously published microarray (Katsarou et al., 2016a).

Annotation name	Sol genomics name	MSAP-Seq results		Microarray results	
		Log2FC	Padja	Log2FC	Padja
Niben101Scf08028g00010.1	E3 ubiquitin-protein ligase TRIM39	3,801661475	0,001854042	0,40164912	0.013975593798084
Niben101Scf07477g02005.1	Receptor-like protein kinase	-2,679645836	0,012974215	-0,3245364	0.0437464692366639

## 4 Discussion

DNA methylation is an important endogenous mechanism widespread across all eukaryotes resulting in epigenetic alterations. These changes in plants have been associated with gene expression, imprinting and stability (Zhang et al., 2018; Erdmann and Picard, 2020; Gallego-Bartolome, 2020; Kryovrysanaki et al., 2022; Lucibelli et al., 2022). Plant DNA methylation occurs in three patterns, CG, CHG and CHH where H represents A, C or T. Even though it is not very clear how, methylation ‘hot spots’ exists within the genome for instance in TE and repetitive DNA regions (Quesneville, 2020; Ramakrishnan et al., 2021).

During plant viral infections alterations in DNA methylation may occur either in the host or the viral genome (Raja et al., 2008; Duan et al., 2012; Wang et al., 2018; Diezma-Navas et al., 2019; Sun et al., 2019; Wang et al., 2019; Pérez-Cañamás et al., 2020). These alterations may happen due to a change in an endogenous protein involved in the canonical or non-canonical RdDM mechanism or due to the accumulation of small RNAs. There are several cases of RdDM involvement in symptom development and more generally plant-virus disease resistance (Wang et al., 2005; Raja et al., 2008; Duan et al., 2012; Hamera et al., 2012; Coursey et al., 2018; Wang et al., 2018; Diezma-Navas et al., 2019; Sun et al., 2019; Wang et al., 2019; Leone et al., 2020; Pérez-Cañamás et al., 2020). On the other hand, reports about DNA methylation during viroid infections remain largely contradictory. Viroids were the agents that helped identify the *de novo* methylation mechanism in the 1990s (Wassenegger et al., 1994) and since then have contributed in the discovery of different aspects of the RdDM pathway (Wassenegger and Dalakouras, 2021). Nevertheless, how viroids are using this mechanism remains unclear. PSTVd and HLVd have been suggested to induce hypermethylation in potato and hop plants respectively (Lv et al., 2016; Sečnik et al., 2022), whereas HSVd induces hypomethylation in promoters and TE of cucumber and *N. benthamiana* plants (Martinez et al., 2014; Castellano et al., 2015; Castellano et al., 2016a; Castellano et al., 2016b). In addition, upon co-infection of PSTVd and a geminivirus, hypermethylation of viral DNA was observed while during a triple co-infection in hop plants with CBCVd, HLVd and HSVd, genomic hypomethylation was reported (Torchetti et al., 2016; Sečnik et al., 2022). Since viroids do not encode for proteins, any effect on DNA methylation could result from the activity of DCL produced vd-siRNAs that bind methylation-related AGO proteins and therefore potentially activate RdDM pathways (Dadami et al., 2013; Minoia et al., 2014; Katsarou et al., 2016a; Katsarou et al., 2022). Such a correlation has already been shown for CMV and various tobamoviruses (Wang et al., 2018; Leone et al., 2020). Nevertheless, other non-DCL dependent mechanisms could also be involved (Cuerda-Gil and Slotkin, 2016).

To investigate if there are any methylation alteration during viroid infection related to siRNAs, we analyzed already published data of HTS from healthy and PSTVd-infected plants (2 and 4 wpi) (Di Serio et al., 2010; Navarro et al., 2021), bioinformatically isolated the 24 nt population and identified possible targets of these siRNAs in endogenous promoters and TEs of *N. benthamiana*. We found only a few targets with different population sizes of 24 nt theoretically binding to these sequences and chose one promoter (germin-like) and one TE (LTR/Copia) for further analysis. We used the promoter of cellulose synthase as a positive control since it has been shown before to be hypomethylated in potato PSTVd-infected plants (Lv et al., 2016). For the analysis, we used Chop-PCR with various MSREs combined with a sophisticated imaging system for results quantification (Dasgupta and Chaudhuri, 2019). None of the enzymes used identified significant differences in healthy and infected *N. benthamiana* plants. It is noteworthy that no methylation differences were observed even in the promoter of cellulose synthase, our positive control. This is possibly due to the different plant species compared (Lv et al., 2016). Since we obtained only negative results, we reasoned that we may have missed differentially methylated targets due to sub-optimal target selection. Additional targets selected to address the issue of target selection again showed no changes of their methylation pattern following viroid infection. Subsequently, we hypothesized that potential inefficiencies in enzyme cleavage during Chop-PCRs could mask small methylation changes, and therefore performed Bisulfite Sequencing for tested targets cellulose synthase, germin-like and TE targets. Once more, no differences in the methylation in CG, CHG or CHH patterns upon PSTVd infection was observed, which suggested that the increase of 24 nt population binding in these targets did not seems interfere with methylation.

Nevertheless, we reasoned that if the genome of *N. benthamiana* is highly methylated, we may not observe any significant difference due to the buffering of the system. Since in *A. thaliana* dcl3 knock-out has been shown to lead to decreased DNA methylation (Xie et al., 2004; Henderson et al., 2006; Stroud et al., 2013), we used a *N. benthamiana* DCL3 knock-down mutant (DCL3.10i) which has a significantly decreased 24 nt population (Katsarou et al., 2016a; Katsarou et al., 2018), and repeated Chop-PCRs in the selected targets. Even with reduced 24 nt siRNAs, no alteration in the methylation status was observed.

All the above techniques used, have in common a limit inherent to arbitrary target selection. In order to understand if PSTVd induces changes in DNA methylation frequency unbiasedly we implemented MSAP-seq, a technique that identifies global methylation patterns. Although MSAP technique has been used for years (Reyna-López et al., 1997; Xiong et al., 1999; Peraza-Echeverria et al., 2001; Salmon et al., 2008; Wang et al., 2011), but rarely combined with HTS. There are of course other existing techniques used to study DNA methylation like whole genome bisulfite sequencing or nanopore sequencing but there are either costly or require sophisticated bioinformatic analysis (Olova et al., 2018; Delahaye and Nicolas, 2021). To our knowledge this is the first time that this technique is used in *N. benthamiana* and the first time that a global methylation technique is used in viroid-infected plants. MSAP-Seq is easy to use, inexpensive, and can provide comprehensive data about methylation coverage. However, the limitation of this technique is the dependency on the cutting activity of used MSRE. In our case, only 9% of the theoretical possible CG sites were identified, suggesting that either *N. benthamiana* genome is highly methylated (both alleles of a given motif)? which is plausible since the CG methylation rate largely differs between species (e.g. 24% in *A. thaliana* and 86% in *Zea mays*), or that the *Hpa*II digestion was inefficient (Cokus et al., 2008; Gent et al., 2013). Furthermore, *N. benthamiana* genome is draft assembled at only a scaffold level therefore the quality of HTS reads mapping may be affected.

The comparison of healthy and PSTVd-infected plants identified only a small amount of differentially methylated positions (DMPs), suggesting that global CG methylation is not a prime mechanism affected upon viroid infection. Nonetheless, we could identify 240 affected DMPs, most were either in intergenic areas (52.61%) or genes (34.14%), and only a small amount was found in promoter regions (4.82%), TEs (2.01%) and repeated regions (6.43%). Nine DMPs were found in more than one location. Gene body and intergenic methylation are usually predominant compared to the other regions although the etiology for this is unclear (Zhang et al., 2006; Zilberman et al., 2007; Zhang et al., 2018). Nevertheless, methylation of these domains is correlated with transcription elongation and cryptic promoters malfunctions (Zhang et al., 2018; Lucibelli et al., 2022).

Looking at the GO enrichment, it was shown that the affected genes are involved in cell integrity (microtubule activity), whereas affected promoters relate to proteins involved in transport. Both of these actions are known to be affected during viral infections hinting to the functional relevance of our results (Niehl et al., 2013). It is noteworthy, that we did not detect differences in the methylation patterns of promoters that had been previously reported to be hypermethylated in PSTVd-infected potato plants (Lv et al., 2016), or in promoters of ribosomal RNAs in HSVd infected cucumber plants (Martinez et al., 2014). This discrepancy could be due to the different pathosystem (plant species and pathogen) used in the two studies, or to the limited resolutive range of our analysis. The latter would suggest that the low number of DMPs we observed might be underestimated. Furthermore, we studied one time point at 3 wpi and there is a possibility that DNA methylation changes could be influenced by the state of infection. This has been shown in a very recent work by Marquez-Mollins et al, where hypomethylation was observed at an early time point and hypermethylation at later time points in HSVd infected cucumbers, suggesting a dynamic status between methylation and infectivity levels (Márquez‐Molins et al., 2023). This implies that even though this work gives a hint in the effect of PSTVd on DNA methylation in *N. benthamiana* plants further investigations are required to fully understand this phenomenon. It is to note that no correlation between the siRNA 24nt populations and the identified targets was observed.

Using previously published microarray results (Katsarou et al., 2016a) we identified two promoter positions with altered CG methylation levels from this study that show respective transcript changes in the microarray data (E3 ubiquitin-protein ligase TRIM39 and Receptor-like protein kinase). It is generally assumed that promoter hypermethylation drives transcript repression and *vice versa* (Zilberman et al., 2007). Recently however, there is an increasing number of reports indicating that hypermethylation can also induce or enhance expression of genes, as it is the case for *ROS1* and genes related to fruit ripening. The mechanism of this is not clear but there are speculations that DNA methylation may influence binding of transcription factors or transcription repressors and change chromatin accessibility (Zhang et al., 2018). This is in accordance with our observations, where one target (Receptor-like protein kinase) presented an hypermethylation correlating with a slight decrease in transcription level, and another target (E3 ubiquitin-protein ligase TRIM39) showed the exact opposite. Both of these genes have been already described as being affected during viroidal (PSTVd) and viral infections (Alcaide-Loridan and Jupin, 2012; Macho and Lozano-Duran, 2019).

Overall, in this study we show that infection of *N. benthamiana* with PSTVd does not lead to extensive changes in DNA methylation frequency since only 1% of the studied CGs were affected. This observation correlates with previously published data where no differences in the expression of proteins involved in the RdDM pathways were observed during PSTVd infection in *N. benthamiana* (Katsarou et al., 2016a; Katsarou et al., 2022). However, in tomato plants it has been showed that PSTVd induces the expression of all genes involved in methylation (Torchetti et al., 2016), thus suggesting a different plant response to PSTVd infection depending on the host. Furthermore, our analysis is focusing mostly in CGs, leaving open the possibility that methylation of the other patterns (CHG or CHH) occurs, even though the analysis of the few targets presented in this study doesn’t support this. Nevertheless, we cannot exclude that even small CG methylation differences are capable of affecting phenotype and viroid infectious cycle, as has been demonstrated in cases like epitranscriptomics (Benoni et al., 2022). Finally, we need to point out that under these experimental conditions we cannot tell if the resulting effect is beneficial for the viroid’s biological cycle or if it is just the outcome of the stress caused by infection. Yet, this is the case for most published studies and for the moment there is no way to distinguish between these two possibilities.

Viroid and more generally circular non-coding RNA research is a productive field with an increasing number of reports the last few years (Cervera and de la Peña, 2020; Forgia et al., 2023). Understanding the mechanisms involved in viroid biological cycle will pave the way to elucidate how these interesting pathogens despite their simplicity manage to be so successful in nature.

## Data availability statement

The datasets presented in this study can be found in online repositories. The names of the repository/repositories and accession number(s) can be found below: https://www.ebi.ac.uk/ena, PRJEB64208.

## Author contributions

MT: Investigation, Writing – review & editing. NB: Writing – review & editing, Data curation. PK: Investigation, Writing – review & editing. CA: Data curation, Writing – review & editing. MC: Data curation, Writing – review & editing. BN: Conceptualization, Investigation, Methodology, Writing – review & editing. ML: Data curation, Supervision, Writing – review & editing, Methodology. FD: Conceptualization, Methodology, Writing – review & editing. KrK: Funding acquisition, Methodology, Supervision, Writing – review & editing. KoK: Conceptualization, Investigation, Methodology, Supervision, Writing – original draft, Writing – review & editing.

## Supplementary material

Supplementary Figure 1 Observed phenotype for *N. benthamiana* wt and DCL3.10i non-infected and PSTVd-infected plants.

Supplementary Figure 2
*Mcr*BC experiments in non-infected and PSTVd infected *N. benthamiana* plants. Two replications are presented here for targets **(A)** control choloplastic DNA **(B)** Cellulose synthase, **(C)** germin-like, **(D)** TE, **(E)** ribosomal L18a, **(F)** vironine synthase, **(G)** vacuolar protein sorting-associated protein 1 and **(H)** glucan endo 1-3beta-glucosidase.

Supplementary Figure 3
*Hpa*II and *Nla*III experiments in non-infected and PSTVd-infected *N. benthamiana* plants. Only a few targets with specific sites recognized by these MSRE are presented. Two replications are presented here for targets germin-like (gl), vironine synthase (vs), vacuolar protein sorting - associated protein (vtaI), cellulose synthase (cs), ribosomal L18a (L18a) and TE. The remaining replicates is presented in and.

Supplementary Figure 4
*Mcr*BC **(A)**, *Hpa*II **(B)** and *Nla*III **(C)** digestions in DNA of DCL3.10i non-infected and PSTVd-infected plants. Two replications are presented here for control DNA (chl) and for targets cellulose synthase (cs), germin-like (gl), TE, ribosomal L18a (L18a), vironine synthase (vs), vacuolar protein sorting - associated protein (vtaI) and glucan endo 1-3 beta – glucosidase (1-3 beta).

Supplementary Figure 5 Representation of the number and location of reads at a CG position of selected targets in non-infected (NI) and PSTVd infected (INF) conditions. **(A)** Aquaporine-4, **(B)** MATE, **(C)** PLATZ using Track data in JBrowser of SolGenomics (https://solgenomics.net/jbrowse_solgenomics/).

Supplementary Figure 6 Analysis of the biological process of genes bodies **(A)** and promoter **(B)** influenced during PSTVd infection in *N. bethamiana* plants using PlantRegMap.
