# Supplementary figures and images for: PSTVd infection in Nicotiana benthamiana plants has a minor yet detectable effect on CG methylation
Source: Front Plant Sci. 2023 Oct 31;14:1258023. doi: 10.3389/fpls.2023.1258023 (PMC10645062; doi:10.3389/fpls.2023.1258023)

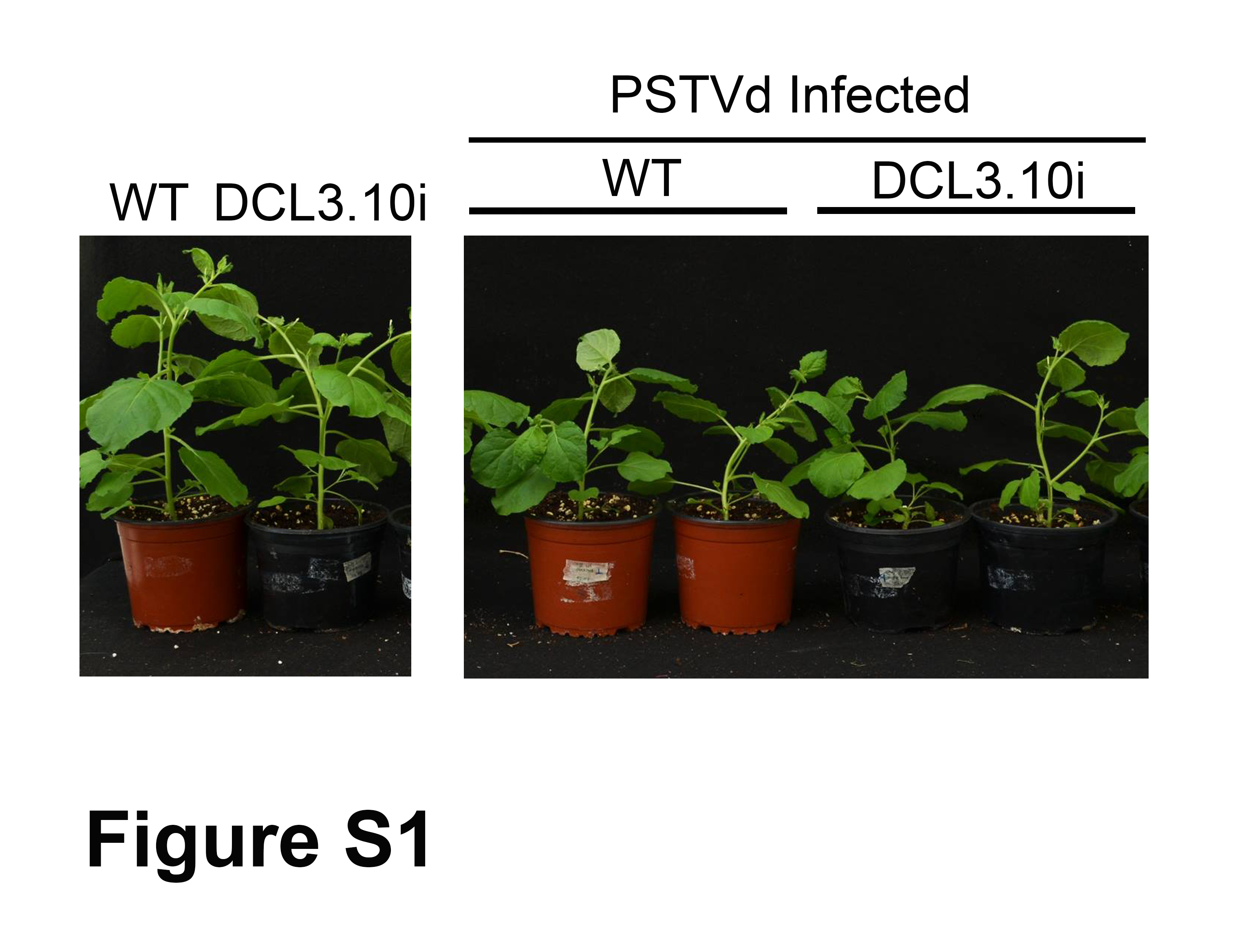

Supplement: Supplementary Figure 1 — Observed phenotype for N. benthamiana wt and DCL3.10i non-infected and PSTVd-infected plants. [file Image_1.tif]

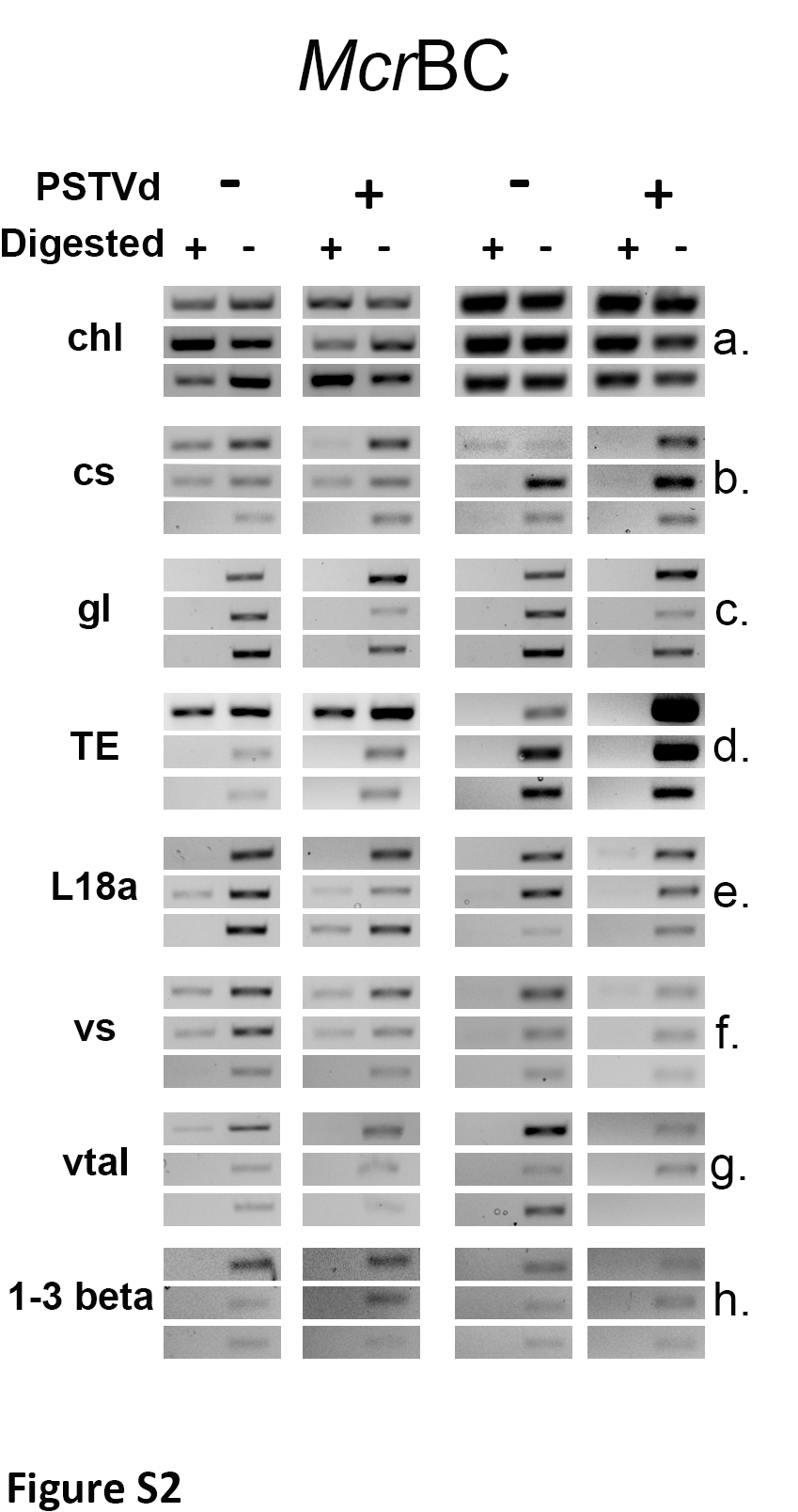

Supplement: Supplementary Figure 2 — McrBC experiments in non-infected and PSTVd infected N. benthamiana plants. Two replications are presented here for targets (A) control choloplastic DNA (B) Cellulose synthase, (C) germin-like, (D) TE, (E) ribosomal L18a, (F) vironine synthase, (G) vacuolar protein sorting-associated protein 1 and (H) glucan endo 1-3beta-glucosidase. [file Image_2.tif]

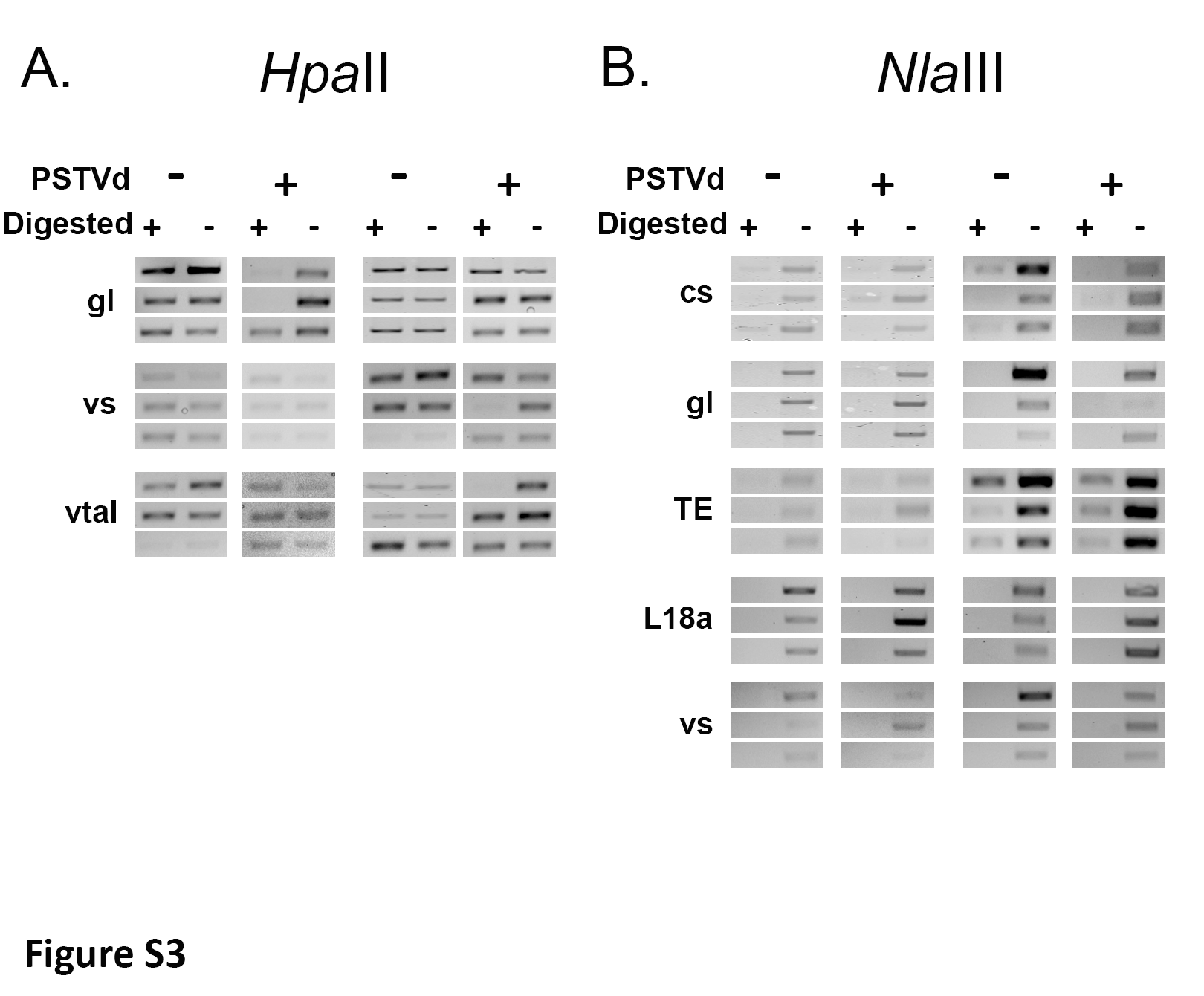

Supplement: Supplementary Figure 3 — HpaII and NlaIII experiments in non-infected and PSTVd-infected N. benthamiana plants. Only a few targets with specific sites recognized by these MSRE are presented. Two replications are presented here for targets germin-like (gl), vironine synthase (vs), vacuolar protein sorting - associated protein (vtaI), cellulose synthase (cs), ribosomal L18a (L18a) and TE. The remaining replicates is presented in and. [file Image_3.tif]

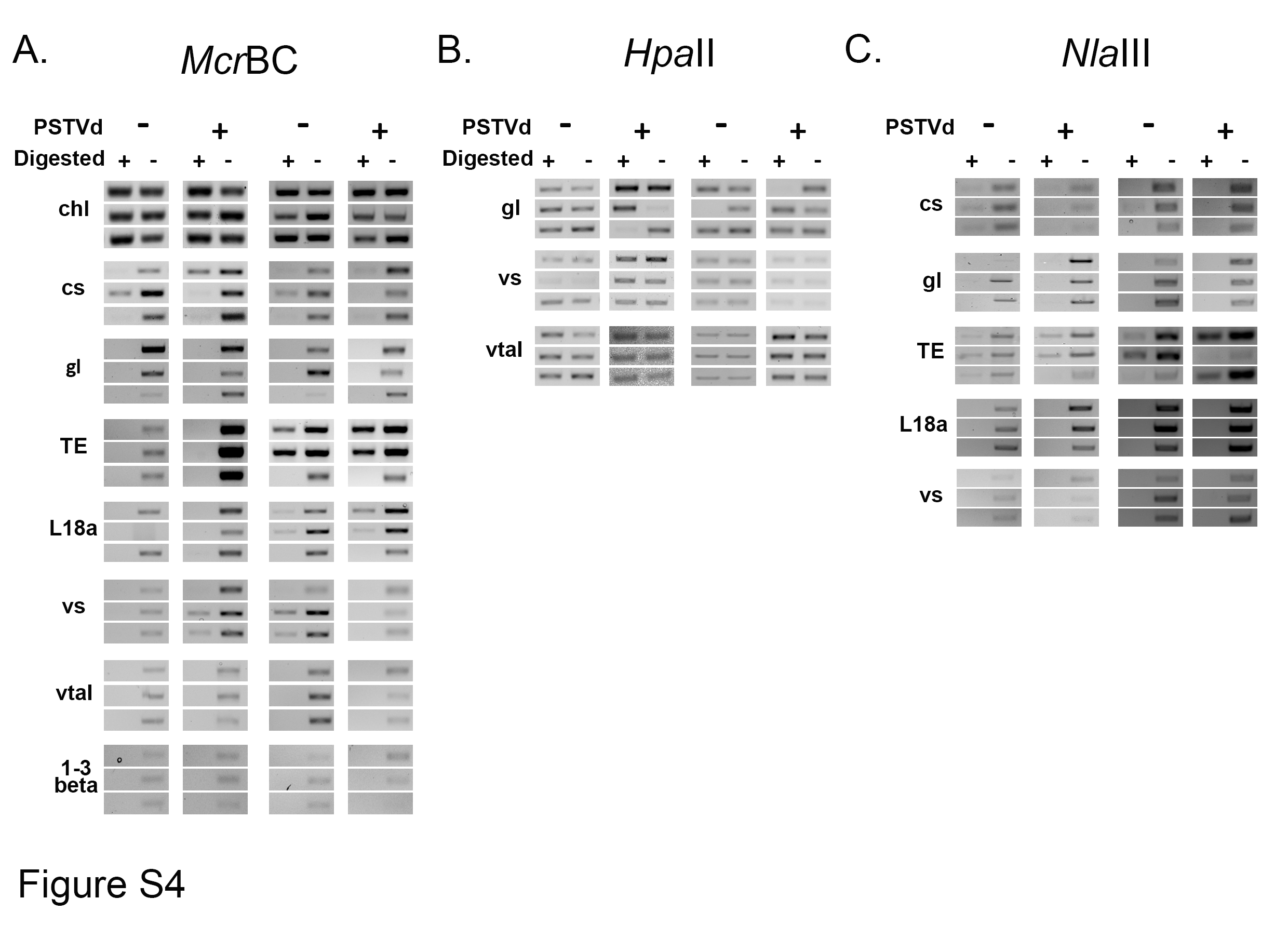

Supplement: Supplementary Figure 4 — McrBC (A), HpaII (B) and NlaIII (C) digestions in DNA of DCL3.10i non-infected and PSTVd-infected plants. Two replications are presented here for control DNA (chl) and for targets cellulose synthase (cs), germin-like (gl), TE, ribosomal L18a (L18a), vironine synthase (vs), vacuolar protein sorting - associated protein (vtaI) and glucan endo 1-3 beta – glucosidase (1-3 beta). [file Image_4.tif]

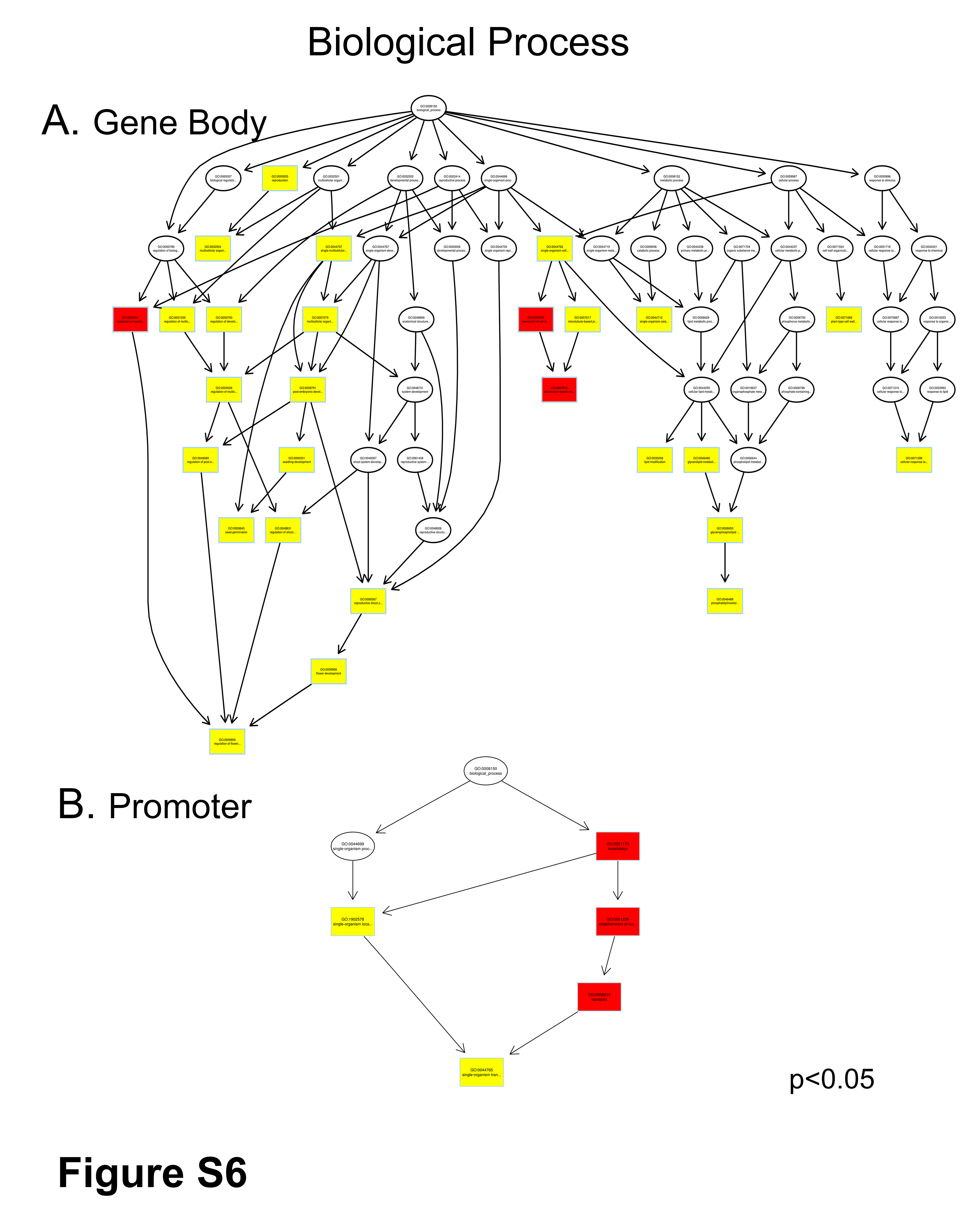

Supplement: Supplementary Figure 6 — Analysis of the biological process of genes bodies (A) and promoter (B) influenced during PSTVd infection in N. bethamiana plants using PlantRegMap. [file Image_6.tif]
