# Supplementary material for: PSTVd infection in Nicotiana benthamiana plants has a minor yet detectable effect on CG methylation
Source: Front Plant Sci. 2023 Oct 31;14:1258023. doi: 10.3389/fpls.2023.1258023 (PMC10645062; doi:10.3389/fpls.2023.1258023)
Supplement: Supplementary file 8 [file Table_2.docx]

| **Name** | **Sequence**  **(5’-3’)** | **Annealing temperature (^o^C)** | **Purpose** | **Reference**  **(If any)** |
| --- | --- | --- | --- | --- |
| Nb_chloro_FW | ATGGGGAAGGATGGATTGTTAGTGT | 56 | MSRE-PCR/ Bisulfite-sequencing | present work |
| Nb_chloro_RV | TTTCTCCTCCAAAAATAACTTCTCC | 56 | MSRE-PCR/ Bisulfite-sequencing | present work |
| Nb_cellulose synthase_FW | AGATAAAAAAAAAGAAATATGATGG | 49 | MSRE-PCR/ Bisulfite-sequencing | present work |
| Nb_cellulose synthase_RV | TCTCCCTCTTTTACTRTTTCAA | 49 | MSRE-PCR/ Bisulfite-sequencing | present work |
| Nb_germin like_FW | TTGGGATAGTTCCTTGGTCC | 54 | MSRE-PCR | present work |
| Nb_germin like_RV | TTGGGGATTACGACCTCTGT | 54 | MSRE-PCR | present work |
| Nb_transposon_FW | AAAAGGTAATTGATTATTTAAAGAAGG | 51 | MSRE-PCR/ Bisulfite-sequencing | present work |
| Nb_transposon_RV | TCCCTTTTARATACCRCAATA | 51 | MSRE-PCR/ Bisulfite-sequencing | present work |
| Nb_BS_germin like_FW | TTATGTTTYAAGTAATTTTTGAAGT | 59 | Bisulfite-sequencing | present work |
| Nb_germin like_BS_RV | ATRTTCTTCTATCTAACTACTTTAA | 59 | Bisulfite-sequencing | present work |
| Nb_vtaI_FW | YTYTTGTATATAGTAGAAAAGTTTTA | 54 | MSRE-PCR | present work |
| Nb_vtaI_RV | ACTAAAATAAATACTTAACTAAAATTTTC | 54 | MSRE-PCR | present work |
| Nb_vironine synthase_FW | CCCTTTACAAAGTGATGAGGATAC | 54 | MSRE-PCR | present work |
| Nb_vironine synthase_RV | TATGTCCATAATTTCGGATTAGGC | 54 | MSRE-PCR | present work |
| Nb_endo glucan_FW | GACCTTTCACTATTCAGTTACGGT | 54 | MSRE-PCR | present work |
| Nb_endo glucan_RV | GCCCTGGATCTTAGGAAAACAGAG | 54 | MSRE-PCR | present work |
| Nb_ribosomal_FW | CGCATGCAGTTGATTGCAAG | 54 | MSRE-PCR | present work |
| Nb_ribosomal_RV | CACAGAAGACTACGCTCAACTC | 54 | MSRE-PCR | present work |
| MSAP_EcoRI_ A1 | CTCGTAGACTGCGTACC | 95/56 | Adapter *Eco*RI/  MSAP_PCR_primer | [1] |
| MSAP_EcoRI_ A2 | AATTGGTACGCAGTCTAC | 95 | Adapter *Eco*RI | [1] |
| MSAP_HpaII_A1 | GACGATGAGTCTAGAA | 95/56 | Adapter *Hpa*II /MSAP_PCR_primer | [1] |
| MSAP_HpaII_A2 | CGTTCTAGACTCATC | 95 | Adapter *Hpa*II | [1] |
| MATE1_BS_FW | ATAGGYGAGAYGGAGAAT | 50 | Bisulfite-sequencing | present work |
| MATE1_BS_RV | AATTCACCCCACCATTTCTTT | 50 | Bisulfite-sequencing | present work |
| PLATZ_BS_FW | AAGGTGTATTAYTGTTTTATGTGA | 50 | Bisulfite-sequencing | present work |
| PLATZ_BS_RV | CTARAATCCTAAACCCRATAACAT | 50 | Bisulfite-sequencing | present work |
| Aquaporin_BS_FW | TATGAGAATAGTTGAATAGAAGTTT | 51 | Bisulfite-sequencing | present work |
| Aquaporin_BS_RV | CCCAACRTCCATTCTCATCAT | 51 | Bisulfite-sequencing | present work |
| M13_RV | CAGGAAACAGCTATGAC | 55 | Sequencing of plasmids | universal primer |

**Table S2: Primers used in this study**

**Supplemental Bibliography for Table S2**

1. Chwialkowska, K., Korotko, U., Kosinska, J., Szarejko, I. and Kwasniewski, M., 2017. Methylation Sensitive Amplification Polymorphism Sequencing (MSAP-Seq)—A Method for High-Throughput Analysis of Differentially Methylated CCGG Sites in Plants with Large Genomes. *Frontiers in Plant Science*, 8.
